# Neurodevelopmental disorders in pediatric patients with atopic dermatitis: a systematic review and meta-analysis

**DOI:** 10.3389/fmed.2026.1975921

**Published:** 2026-10-29

**Authors:** Hong-Fei Wang, Chen-Xi Ma, Zi-Jie Chen, Zheng Lin, Hao-Nan Wang, Yi Cao, Qiu-Shuang Li, Hong-Bin Luo

**Affiliations:** 1 The First Affiliated Hospital of Zhejiang Chinese Medical University (Zhejiang Provincial Hospital of Chinese Medicine), Hangzhou, China; 2 The First School of Clinical Medicine, Zhejiang Chinese Medical University, Hangzhou, China; 3 Graduate School, Tianjin University of Traditional Chinese Medicine, Tianjin, China

**Keywords:** atopic dermatitis, neurodevelopmental disorders, prevalence, systematic review, meta-analysis

## Abstract

**Background:**

Neurodevelopmental disorders (NDs) are increasingly recognized among children with atopic dermatitis (AD). This systematic review and meta-analysis aimed to summarize the prevalence of NDs in pediatric patients with AD and quantify the associations between AD and specific neurodevelopmental outcomes.

**Methods:**

We searched Embase, PubMed, Cochrane Library, Web of Science, and PsycINFO from their inception to 1 December 2025. Observational studies reporting adjusted estimates and/or prevalence for NDs in pediatric patients with AD were included. Exploratory subgroup analyses were undertaken to ascertain sources of heterogeneity. Funnel plots and Egger's test were used to assess publication bias.

**Results:**

From 2,514 initial records, 39 studies reporting ND outcomes in pediatric patients with AD were included. The pooled prevalence rates for NDs were as follows: attention-deficit/hyperactivity disorder (ADHD, 6.01%), autism spectrum disorder (ASD, 0.81%), learning disability (LD, 11.34%), conduct disorder (CD, 2.76%), and peer problems (5.98%). AD was associated with higher odds of ADHD (OR = 1.34, 95% CI, 1.21–1.50), ASD (OR = 2.11, 95% CI, 1.07–4.16), LD (OR = 1.71, 95% CI, 1.41–2.06), intellectual disability (OR = 1.39, 95% CI, 1.33–1.45), and CD (OR = 1.35, 95% CI, 1.16–1.58), but not peer problems (OR = 1.10, 95% CI, 0.96–1.26). HR-based estimates for ADHD (HR = 1.54, 95% CI, 0.97–2.44) and ASD (HR = 2.09, 95% CI, 0.54–8.19) were directionally similar but less precise. Substantial heterogeneity was present across several analyses.

**Conclusion:**

Pediatric AD was associated with higher odds of several neurodevelopmental outcomes compared with children without AD. However, substantial heterogeneity, potential detection bias, and the observational nature of the available evidence limit causal interpretation. Further well-designed longitudinal studies using standardized exposure and outcome definitions, adequate control of confounding, and clearer temporal characterization are needed to clarify the consistency and directionality of these associations.

**Systematic Review Registration:**

https://www.crd.york.ac.uk/PROSPERO/view/CRD42024545405, PROSPERO CRD42024545405.

## Background

Atopic dermatitis (AD) is a common chronic pruritic inflammatory skin disease affecting about 20% of children and adolescents (1). The latest data from the Global Burden of Disease 2019 indicated that AD continues to cause significant global morbidity from 1990 to 2019 (2). For children, neurodevelopmental disorders (NDs) and sleep disorders are prevalent comorbid conditions associated with AD. NDs represent a heterogeneous group of conditions that emerge early in life and often persist throughout the lifespan (3), among which attention-deficit/hyperactivity disorder (ADHD), autism spectrum disorder (ASD), learning disability (LD), and intellectual disability (ID) are common diagnoses. From 2018 to 2021, the prevalence of any NDs among children aged 3–17 years in the US was high at 16.65%, with a significant increase in co-occurring LD and ID (4).

Underpinned by shared genetic markers and underlying pathophysiological mechanisms (systemic inflammation and immune dysregulation), AD and NDs may mutually exacerbate each other, perpetuating a vicious cycle that persists into adulthood (5–7). Recent research has highlighted a consistent annual rise in the prevalence of NDs among individuals with AD compared with those without the condition, accompanied by a substantially higher risk of developing these conditions (8–10). This has drawn considerable attention from both the public and clinical communities.

Although existing research has addressed the association between AD and NDs, the literature has predominantly focused on ADHD and ASD, with comparatively limited attention given to LD, ID, and other NDs (11–13). Furthermore, the reported prevalence of NDs in patients with AD varied significantly (14–16), potentially due to differences in study populations (e.g., disease severity and ethnicity) and diagnostic methods. In addition, a growing number of studies have shown that some variables, such as the severity of AD, sleep disorders, allergic history, and maternal conditions during pregnancy, have been associated with neurodevelopmental outcomes (12, 17–19). However, results remain inconsistent, and no research has systematically compiled and quantitatively evaluated these associated factors. To address this important gap, we aimed to summarize the prevalence of NDs in pediatric patients with AD, quantify the associations between AD and specific neurodevelopmental outcomes, and explore potentially associated factors.

## Methods

This systematic review followed the Preferred Reporting Items for Systematic Reviews and Meta-Analyses (PRISMA) guidelines (20). The protocol was pre-registered in PROSPERO (CRD42024545405).

### Search strategy

Two reviewers independently searched Embase, PubMed, Web of Science, Cochrane Library, and PsycINFO from their inception to 1 December 2025. The search strategy incorporated subject phrases and free-text terms, including “atopic dermatitis”, “neurodevelopmental disorders”, “attention deficit hyperactivity disorder”, “autism spectrum disorder”, “intellectual disability”, “learning disabilities”, “tic disorders”, “conduct disorder”, “oppositional defiant disorder”, and their associated variations. The full search strategy is detailed in Supplementary Table S1. To enhance the thoroughness of our search, we manually screened the reference lists of every relevant study identified in the initial search for additional pertinent research.

### Eligibility and exclusion criteria

Eligible studies were required to be peer-reviewed original articles published in English and of observational design, including cohort, case–control, or cross-sectional studies. Studies were required to provide data relevant to the following PECO (Population, Exposure, Comparator, Outcome): (1) Population: pediatric individuals from birth to 18 years of age; (2) Exposure: presence of AD; (3) Comparator: pediatric individuals without AD for association analyses, whereas a comparator was not required for prevalence analyses; (4) Outcome: The primary neurodevelopmental outcomes were ADHD, ASD, LD, and ID. Other reported neurodevelopmental outcomes were considered secondary outcomes when relevant data were available. AD and NDs were ascertained using clinical diagnoses, diagnostic codes or medical records, physician-confirmed or caregiver-reported physician diagnoses, or validated standardized questionnaires. No restrictions were imposed by sex, race, or country.

Exclusion criteria were as follows: (1) studies from which relevant data could not be extracted; (2) case reports, reviews, letters or conference/meeting abstracts without original data, and commentaries.

### Data extraction and quality assessment

Two researchers independently extracted data from the included articles, with subsequent cross-checking to ensure accuracy. The extracted data encompassed the first author, publication year, study period, study design, country, total AD sample size, sample source, methods of assessment for AD and NDs, and outcome measures (reported prevalence and effect estimates). For studies using complex survey designs, unweighted analytic sample sizes were extracted when available, whereas survey-weighted population estimates were not treated as participant counts or meta-analytic denominators. For AD as an exposure for NDs, adjusted odds ratios (ORs), relative risks (RRs), hazard ratios (HRs), and associated 95% confidence intervals (CIs) were extracted. No additional information was sought from the original authors.

The quality of each cohort or case–control study was assessed by the same reviewers using the Newcastle–Ottawa Quality Assessment Scale (NOS) (21), while the Agency for Healthcare Research and Quality (AHRQ) guidelines were applied to evaluate the quality of cross-sectional studies (22). Any disagreements were resolved by a third reviewer.

### Statistical analysis

Two reviewers independently conducted statistical analyses using Stata version 18.0 (StataCorp, LLC, College Station, TX, USA, authorized by The First Affiliated Hospital of Zhejiang Chinese Medical University). We pooled the following outcomes according to feasibility assessment: (1) the prevalence of NDs in pediatric patients with AD; (2) the association between AD and NDs; and (3) factors associated with NDs among pediatric patients with AD. When studies used overlapping databases, only one representative estimate was retained in each pooled analysis to avoid double-counting participants. Priority was given to studies with broader population coverage, more appropriate outcome ascertainment, and more fully adjusted estimates. The same deduplication rule was applied to subgroup analyses. For prevalence analyses, only eligible cross-sectional and cohort studies were included. Case–control studies were excluded because they do not provide unbiased prevalence estimates. For studies reporting unweighted data, the numerator was the number of children with AD who had the ND of interest, and the denominator was the number of children with AD in the corresponding analytic sample. For complex survey studies, reported survey-weighted prevalence estimates and their 95% CIs were used to derive study-specific effect estimates and standard errors without reconstructing event counts from weighted population estimates. Prevalence estimates were logit-transformed and pooled using inverse-variance methods, and the pooled estimates were subsequently back-transformed to proportions. For meta-analyses with sufficient studies, we explored heterogeneity in prespecified exploratory subgroup analyses of geographical region, study design, sample source (population- and registry-based studies vs. clinical sample-based studies), sample size, and assessment of NDs. Subgroup differences were assessed using interaction tests, and a *P* < 0.05 was considered statistically significant. Given the anticipated between-study heterogeneity from differences in analytical methods and population characteristics, random-effects models were used to pool the effect estimates, with the heterogeneity variance *τ*^2^ estimated using the restricted maximum likelihood (REML) (23). For association analyses, adjusted ORs, RRs, and HRs were synthesized separately on the logarithmic scale using inverse-variance methods when at least two studies reported the same effect measure. Standard errors were derived from the reported 95% CIs as [ln(upper CI) − ln(lower CI)]/3.92, and pooled estimates were subsequently exponentiated for presentation. When a study reported more than one type of effect measure, each measure was considered only within its corresponding effect measure-specific analysis. Heterogeneity was assessed using Cochran's *Q*-test, with substantial heterogeneity defined as *I*^2^  > 50% or *P* < 0.1 in the *Q*-test (24). For meta-analyses including at least five studies, 95% prediction intervals (PI) were calculated when substantial heterogeneity was present (25, 26). Leave-one-out sensitivity analyses were performed when sufficient studies were available and were not undertaken for analyses with very few studies. Funnel plots and Egger's regression were used to evaluate publication bias in meta-analyses with 10 or more studies. Significant publication bias was indicated by an asymmetric funnel plot or *P* < 0.05 (27). If significant publication bias was detected, we conducted the trim-and-fill procedure to identify potential “missing studies” and investigate their impact on the pooled effect estimates. When data could not be combined due to substantial clinical or methodological heterogeneity or insufficient studies, the results were reported qualitatively.

## Results

### Study selection

From PubMed (*n* = 297), Embase (*n* = 1,128), Cochrane Library (*n* = 19), Web of Science (*n* = 818), and PsycINFO (*n* = 252), a total of 2,514 records were found. After removing duplicates (*n* = 565) and excluding studies that did not meet the criteria (*n* = 1,880), 39 studies were ultimately included (Figure 1).

**Figure 1:**
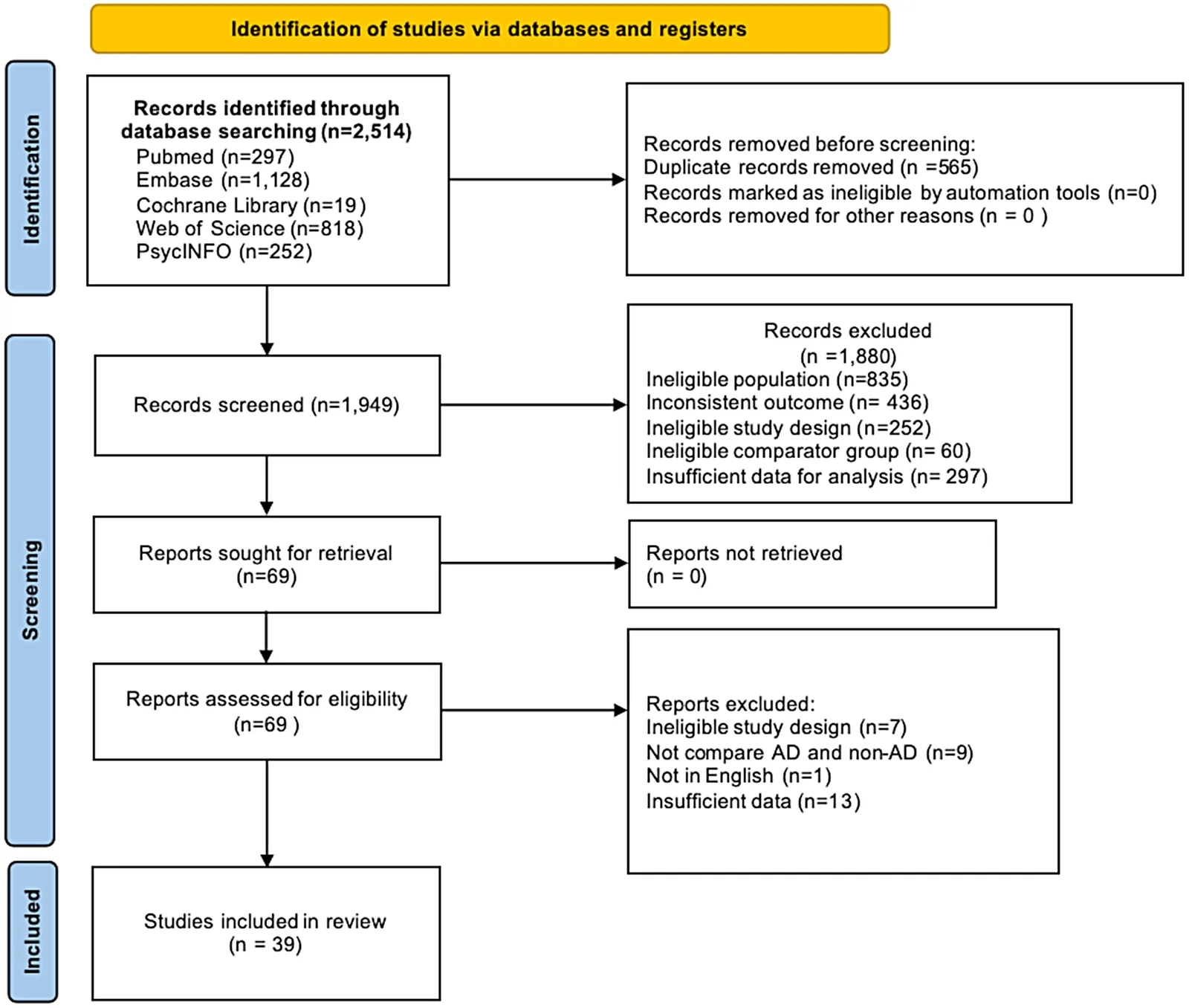
PRISMA flow chart.

### Characteristics of the included studies

A total of 39 studies, consisting of 27 cross-sectional studies, 11 cohort studies, and 1 case–control study, are summarized in Table 1. These studies were carried out in Europe (*n* = 12), Asia (*n* = 14), and North America (*n* = 13) and were published between 2009 and 2024. NOS scores ranged from 7 to 9 among the cohort and case–control studies, whereas AHRQ scores ranged from 5 to 8 among the cross-sectional studies. Details are available in Supplementary Table S2.

**Table 1:** Characteristics of participants and studies.

Study	Country	Study type	Study period	AD sample size	Data source	Assessment method		Outcome measure(s)	
						AD	NDs	Prevalence (%)	Effect estimates (95% CI)
Ahn et al. (28)	Korea	Cross-sectional	2002–2014	24,907	2015 Korean National Health Insurance Research Database	ICD 10 code L20.9	ICD 10 codes	ADHD: 0.87	NA
								ASD: 0.24	
								CD: 0.04	
Augustin et al. (29)	Germany	Cross-sectional	2009	30,354	German statutory health insurance company (Gmünder Ersatzkasse)	ICD 10 code L20	ICD 10 codes	ADHD: 8.11	ADHD: OR 1.36 (1.30−1.42)
Ballardini et al. (48)	Russia	Cohort	1996–2005	11,668	PROBIT	ISAAC questionnaire	SDQ	NA	ADHD: OR 0.91 (0.77−1.08)
									CD: OR 1.12 (0.95−1.31)
									Peer problems: OR 0.87 (0.70–1.08)
Boemanns et al. (13)	Germany	Cross-sectional	May 2003–May 2006	1,164	2003–2006 KiGGS	Parental report of physician diagnosis	Parental report of physician diagnosis	ADHD: 8.10	ADHD: OR 1.69 (1.25–2.29)
Catal et al. (30)	Turkey	Cross-sectional	July 2013–January 2014	80	The Pediatric Allergy and Asthma Clinics of Inonu University	Hanifin and Rajka classification	ECI-4	ADHD: 23.8	ADHD: OR 2.57 (1.05–6.30)
								TD: 6.3	
								ODD:21.3	
								CD: 7.5	
Cheng et al. (61)	USA	Cross-sectional	1996–2015	2,553	1996–2015 MEPS	ICD-9-CM	CIS	NA	Peer problems: OR 1.17 (1.05–1.31)
Feng et al. (31)	China	Cross-sectional	October 2018–October 2019	89	Children's Hospital of Chongqing Medical University	UK Working Party's Diagnostic Criteria	SNAP-IV questionnaire	ADHD: 10.1	NA
								ODD: 5.6	
Fishbein et al. (32)	USA	Cross-sectional	May 2019–July 2019	180	National Eczema Association and Op4G	Parent/self-report	Parent/self-report	ADHD: 16.67	NA
Genuneit et al. (33)	Germany	Cohort	November 2000–November 2001	114	Ulm University birth cohort	Parental report of physician diagnosis and/or physicians' reports	Parental report of physician diagnosis	ADHD: 10.5	ADHD: RR 5.17 (2.18–12.28)
Horev et al. (49)	Israel	Case–control	NA	840	CHS	Hospital-based medical records	Hospital-based medical records	ADHD: 7.1	ADHD: OR 2.1 (1.3–3.4)
Hou and Silverberg (12)	USA	Cross-sectional	1997–2018	23,353	1997–2018 US NHIS	Survey questionnaire of the NHIS	Survey questionnaire of the NHIS	ADHD: 10.0	ADHD: OR 1.36 (1.27–1.46)
								ASD: 1.67	ASD: OR 1.52 (1.29–1.78)
Hsu et al. (34)	USA	Cross-sectional	2002–2012	NA	2002–2012 NIS	ICD-9-CM code 691.8	ICD-9-CM and DRG codes	ADHD: 2.06^a^	ADHD: OR 1.14 (0.95–1.36)
								Developmental disorders: 1.87^a^	Developmental disorders: OR 1.23 (1.06–1.44)
Huang et al. (50)	USA	Cross-sectional	January 2017–December 2017	86,969	IBM	ICD 10 code L20	ICD 10 codes	NA	ADHD: OR 1.11 (1.06–1.16)
									Developmental/intellectual disabilities: OR 1.39 (1.33–1.45)
Huang et al. (45)	USA	Cross-sectional	2005–2018	1,376	2005–2018 US NHIS	Parental report of physician diagnosis	Parental report of physician diagnosis	ADHD: 13.66	ADHD: OR 1.45 (1.06–1.99)
								ASD: 2.69	ASD: OR 1.89 (1.11–3.22)
								LD: 16.13	LD: OR 1.66 (1.26–2.18)
								ID: 3.05	ID: OR 1.24 (0.74–2.09)
Jackson-Cowan et al. (47)	USA	Cross-sectional	2008–2018	13,398	2008–2018 US NHIS	Self-report of physician diagnosis	Self-report of physician diagnosis	ADHD: 10.78	ADHD: OR 1.31 (1.20–1.42)
								Developmental delay: 6.96	Developmental delay: OR 1.54 (1.40–1.70)
								Self-reported memory impairment: 0.87	Self-reported memory impairment: OR 1.84 (1.34–2.51)
Johansson et al. (35)	Sweden	Cohort	February 1994–December 1996 (follow-up 16 years)	1,178	Swedish BAMSE birth cohort and Swedish Prescribed Drug Register	Parental questionnaire	Medication record of the Swedish Drug Register	ADHD: 4.90	ADHD: OR 1.12 (0.80–1.56)
Kim et al. (51)	Korea	Cohort	2008–2012	30,557	Korean National Health Insurance Service database	ICD 10 code L20.9	ICD 10 codes	NA	ADHD: OR 1.29 (1.07–1.60)
									ID: OR 1.38 (1.04–1.84)
Kuniyoshi et al. (52)	Japan	Cross-sectional	2014–2015	1,641	Japan ToMMo Child Health Study	ISAAC questionnaire	SDQ	NA	ADHD: OR 1.23 (1.04–1.45)
									CD: OR 1.40 (1.19–1.66)
									Peer problems: OR 1.30 (1.09–1.56)
Lee et al. (36)	China	Cohort	January 1998–December 2008	18,473	Taiwan Longitudinal Health Insurance Database 2010	ICD-9-CM code 691 or 691.8	ICD-9-CM code	ADHD: 4.94^b^	ADHD: HR 2.92 (2.48–3.45)
								ASD: 0.99^b^	ASD: HR 8.90 (4.98–15.92)
Liao et al. (37)	China	Cohort	2000–December 2010	387,262	Taiwan NHIRD	ICD-9-CM code 691	ICD-9-CM code	ADHD: 3.7	ADHD: HR 1.16 (1.13–1.19)
								ASD: 0.5	ASD: HR 1.10 (1.03–1.18)
Lin et al. (53)	China	Cross-sectional	2010	251	Taiwan Children Health Study	ISAAC questionnaire	SNAP-26 questionnaire	NA	ADHD: OR 1.66 (1.23–2.25)
									ODD: OR 1.75 (1.08–2.72)
Ma et al. (46)	USA	Cross-sectional	2021	9,223,013^c^	2021 US NHIS	Parental/caregiver questionnaire of physician diagnosis	Parental/caregiver questionnaire of physician diagnosis	Memory difficulty: 11.1^a^	Memory difficulty: OR 1.69 (1.19–2.41)
								LD: 10.8^a^	LD: OR 1.77 (1.28–2.45)
Madulara and Andaya (38)	Philippines	Cross-sectional	NA	206	University of Santo Tomas Hospital Allergy and Immunology Clinic/Doctors' Clinics	NICE guidance	VADPRS	ADHD: 15.05	NA
Romanos et al. (18)	Germany	Cross-sectional	2003–2006	1,952	2003–2006 KiGGS	Medical examination	Medical or psychological examination	NA	ADHD: OR 1.54 (1.24–1.93)
Schmitt et al. (40)	Germany	Cross-sectional	2003–2004	1,436	GKV-database Saxony	ICD 10 code L20	ICD 10 codes	ADHD: 5.15	ADHD: OR 1.47 (1.01–2.15)
Schmitt et al. (15)	Germany	Cohort	1995–1998 (follow-up 10 years)	780	GINIplus	Parental report of physician diagnosis	SDQ	ADHD: 10.0	ADHD: OR 1.25 (0.97–1.61)
								CD: 6.0	CD: OR 1.32 (1.02–1.71)
								Peer problems: 4.0	Peer problems: OR 0.87 (0.63–1.19)
Schmitt et al. (39)	Germany	Cohort	1997–1999 (follow-up 10 years)	367	LISAplus	Parental report of physician diagnosis	SDQ	ADHD: 10.9	ADHD: OR 2.12 (1.34–3.37)
								CD: 6.0	CD: OR 1.71 (0.94–3.10)
								Peer problems: 4.1	Peer problems: OR 0.98 (0.51–1.88)
Shyu et al. (93)	China	Cohort	January 2005–December 2005	10,620	2005 Taiwan NHIRD	ICD 9 code 691	ICD 9 code 314	NA	ADHD: OR 0.73 (0.48–1.09)
Sockler et al. (56)	UK	Cross-sectional	April 1991–December 1992	892	ALSPAC	Parental questionnaire of physician diagnosis	GMDS, WPPSI, WISC, WASI	NA	NA
Strom et al. (17)	USA	Cross-sectional	1997–2013	35,865	1997–2013 US NHIS and 2003/04 and 2007/08 NSCH	Self/caregiver report of physician diagnosis	Self/caregiver report of physician diagnosis	ADHD: 9.40	ADHD: OR 1.14 (1.03–1.26)
Vittrup et al. (41)	Denmark	Cohort	January 1995–December 2012 (follow-up until December 2017)	14,283	Danish nationwide health registries	DNPR diagnostic codes (ICD 10 code L20)	DNPR diagnostic codes (ICD 10 code F90)	NA	ADHD: HR 1.65 (1.33–2.05)
Wan et al. (14)	USA	Cross-sectional	January 2013–December 2017	5,357	2013–2017 US NHIS	Caregiver report	SDQ	ADHD: 14.9	ADHD: OR 1.34 (1.24–1.43)
								CD: 3.1	CD: OR 1.20 (1.10–1.31)
								Peer problems: 12.3	Peer problems: OR 1.20 (1.10–1.30)
Wan et al. (11)	USA	Cross-sectional	January 2013–December 2017	7,284,818^c^	2013–2017 US NHIS	Self-report of physician diagnosis	Self-report of physician diagnosis	LD: 10.4	LD: OR 1.48 (1.28–1.72)
Wan et al. (44)	USA	Cross-sectional	November 2004–November 2019	2,074	PEER	Self-report of physician diagnosis	Self-report of physician diagnosis	LD: 8.2	LD: OR 1.72 (1.11–2.67)
								ADHD: 13.8	
								CD: 5.5	
Wan et al. (42)	UK	Cohort	1994–February 2015	409,431	UK THIN database	THIN diagnostic codes	Diagnosis codes	ADHD: 0.75	ADHD: HR 1.02 (0.97–1.06)
								ASD: 0.71	ASD: HR 1.02 (0.98–1.06)
Wong et al. (54)	China	Cross-sectional	NA	86	Hong Kong Healthy Kids cohort and school-based sample	Parental questionnaire of physician diagnosis	Parental questionnaire of physician diagnosis	NA	ADHD: OR 0.60 (0.20–1.81)
Yaghmaie et al. (16)	USA	Cross-sectional	2007	10,408	2007 NSCH	Parental report of physician diagnosis	Parental report of physician diagnosis	ADHD: 9.57^a^	ADHD: OR 1.87 (1.54–2.27)
								ASD: 2.19^a^	ASD: OR 3.04 (2.13–4.34)
								CD: 6.51^a^	CD: OR 1.87 (1.46–2.39)
Yang et al. (55)	China	Cross-sectional	2010	411	Taiwan CEAS cohort	ISAAC questionnaire	DSM-IV criteria	NA	ADHD: OR 1.71 (0.44–6.60)
Yu et al. (43)	China	Cross-sectional	May 2019–May 2023	80	Zhoushan Branch of Ruijin Hospital	Diagnostic criteria based on clinical practice	SNAP-IV questionnaire	ADHD: 11.25	NA

AD, atopic dermatitis; ADHD, attention-deficit/hyperactivity disorder; ASD, autism spectrum disorder; CD, conduct disorder; ODD, oppositional defiant disorder; LD, learning disability; ID, intellectual disability; TD, tic disorder; No., number; CI, confidence interval; NA, not applicable; HR, hazard ratio; RR, relative risk; OR, odds ratio; DSM, Diagnostic and Statistical Manual of Mental Disorders; ICD, International Classification of Diseases; ICD-9-CM, International Classification of Diseases 9th edition Clinical Modification; ISAAC, International Study of Asthma and Allergies in Childhood; CIS, Columbia Impairment Scale; SDQ, Strengths and Difficulties Questionnaire; DRG, Diagnosis-Related Groups; ECI-4, Early Childhood Inventory-4; SNAP, Swanson, Nolan, and Pelham Rating Scale; NICE, National Institute for Health and Clinical Excellence; VADPRS, Filipino version of the Vanderbilt ADHD Diagnostic Parent Rating Scale; GMDS, Griffiths Mental Development Scales; WPPSI, Wechsler Preschool and Primary Scale of Intelligence; WISC, Wechsler Intelligence Scale for Children; WASI, Wechsler Abbreviated Scale of Intelligence; DNPR, Danish National Patient Register; PROBIT, PROmotion of Breastfeeding Intervention Trial; NHIS, National Health Interview Survey; IBM, IBM MarketScan commercial claims database; KiGGS, German Health Interview and Examination Survey for Children and Adolescents; GINIplus, German Infant Nutrition Intervention plus; LISAplus, influences of lifestyle-related factors on the immune system and the development of allergies in childhood study; NHIRD, National Health Insurance Research Database; CHS, Clalit Health Services; THIN, The Health Improvement Network; NSCH, National Survey of Children's Health; CEAS, Childhood Environment and Allergic Diseases Study; PEER, Pediatric Eczema Elective Registry; ALSPAC, Avon Longitudinal Study of Parents and Children; NIS, Nationwide Inpatient Sample; MEPS, Medical Expenditure Panel Surveys.

a Survey-weighted prevalence estimate.

b Incidence, 1,000 person-years.

c Survey-weighted population estimate; the unweighted AD subgroup sample size was not reported.

### Prevalence rates of NDs in pediatric patients with AD

#### Attention-deficit/hyperactivity disorder

Among pediatric patients with AD, the overall prevalence of ADHD was 6.01% [95% CI, 3.90%–9.16% (12, 13, 15, 16, 28–44), *I*^2^ = 99.89%, *τ*^2^ = 1.11, *P* < 0.001], with a 95% prediction interval of 0.67%–37.88% (Supplementary Figure S1). We conducted exploratory subgroup analyses to examine potential sources of heterogeneity (Table 2). Across geographical regions, the pooled prevalence was highest in North America (8.12%, 95% CI, 3.91%–16.11%), followed by Asia (6.57%, 95% CI, 2.76%–14.81%) and Europe (4.76%, 95% CI, 2.40%–9.23%, Supplementary Figure S2). Studies with smaller sample sizes reported higher prevalence estimates (12.91% vs. 7.41% and 2.78%, Supplementary Figure S3). By study design, the pooled prevalence was 7.88% (95% CI, 4.81%–12.65%) in cross-sectional studies and 3.85% (95% CI, 1.83%–7.91%) in cohort studies (Supplementary Figure S4). Studies based on clinical samples showed a higher pooled prevalence (10.72%, 95% CI, 5.19%–20.83%) than population- or registry-based studies (4.78%, 95% CI, 2.90%–7.79%, Supplementary Figure S5). The pooled prevalence was significantly lower in studies using International Classification of Diseases (ICD) codes (2.27%, 95% CI, 1.19%–4.26%) than in studies using parental, caregiver, or self-reported questionnaires (11.34%, 95% CI, 9.31%–13.75%, Supplementary Figure S6). Johansson et al. (35) identified ADHD using medical records. Significant subgroup differences were observed for sample size and ADHD assessment method (*P* for subgroup differences <0.001), whereas no significant differences were found across geographical region, study design, or sample source.

**Table 2:** Pooled prevalence of neurodevelopmental disorders and exploratory subgroup analyses in pediatric patients with atopic dermatitis.

Subgroups	N	Prevalence (95% CI)	95% prediction interval	Tests of heterogeneity		
				*I*^2^ (%)	*τ* ^2^	*P*-value
ADHD						
Overall prevalence	21	6.01 (3.90–9.16)	0.67–37.88	99.89	1.1061	<0.001
Region (*P* for subgroup differences = 0.563)						
Asia	7	6.57 (2.76–14.81)	0.26–65.79	99.76	1.4439	<0.001
Europe	9	4.76 (2.40–9.23)	0.34–42.43	99.79	1.1640	<0.001
North America	5	8.12 (3.91–16.11)	0.41–65.64	99.80	0.7758	<0.001
Sample size (*P* for subgroup differences < 0.001)						
<1,000	8	12.91 (10.21–16.20)	6.22–24.91	66.77	0.0900	<0.001
1,000–9,999	4	7.41 (4.53–11.88)	0.64–49.83	96.36	0.2718	<0.001
≥10,000	9	2.78 (1.42–5.39)	0.21–28.28	99.95	1.1066	<0.001
Study design (*P* for subgroup differences = 0.109)						
Cross-sectional	13	7.88 (4.81–12.65)	0.96–43.06	99.74	0.9086	<0.001
Cohort	8	3.85 (1.83–7.91)	0.23–40.66	99.91	1.1955	<0.001
Sample source (*P* for subgroup differences = 0.070)						
Clinical sample	6	10.72 (5.19–20.83)	0.68–67.65	96.55	0.8990	<0.001
Population-based/registry-based	15	4.78 (2.90–7.79)	0.51–32.91	99.91	1.0422	<0.001
Assessment of ADHD (*P* for subgroup differences < 0.001^a^)						
ICD codes	8	2.27 (1.19–4.26)	0.20–20.98	99.93	0.8115	<0.001
Medical records	1	4.92 (3.83–6.32)	NA	NA	NA	NA
Parental/caregiver/self-reported questionnaire	12	11.34 (9.31–13.75)	5.45–22.11	95.01	0.1154	<0.001
ASD						
Overall prevalence	6	0.81 (0.42–1.55)	0.07–8.70	99.79	0.6735	<0.001
Region (*P* for subgroup differences < 0.001^a^)						
Asia	3	0.46 (0.24–0.87)	NA	98.32	0.3251	<0.001
Europe	1	0.71 (0.69–0.74)	NA	NA	NA	NA
North America	2	1.99 (1.67–2.36)	NA	75.19	0.0124	0.045
Study design (*P* for subgroup differences = 0.554)						
Cohort	3	0.65 (0.52–0.80)	NA	97.68	0.0326	<0.001
Cross-sectional	3	0.99 (0.24–3.93)	NA	99.61	1.5361	<0.001
Assessment of ASD (*P* for subgroup differences < 0.001)						
ICD codes	4	0.51 (0.31–0.85)	NA	99.57	0.2610	<0.001
Parental/caregiver/self-reported questionnaire	2	1.99 (1.67–2.36)	NA	75.19	0.0124	0.045
LD	3	11.34 (7.41–16.98)	NA	94.06	0.1557	<0.001
CD	6	2.76 (0.67–10.67)	0.01–85.67	99.49	3.1770	<0.001
Peer problems	3	5.98 (2.71–12.68)	NA	95.14	0.4973	<0.001

AD, atopic dermatitis; ADHD, attention-deficit/hyperactivity disorder; ASD, autism spectrum disorder; CD, conduct disorder; LD, learning disability; ICD, International Classification of Diseases; N, number of data points; CI, confidence interval.

a The between-subgroup difference remained statistically significant after excluding the single-study subgroup (medical records for ADHD assessment and Europe for the ASD region, respectively; both *P* for subgroup difference < 0.001).

#### Autism spectrum disorder

Six studies were included in the meta-analysis of ASD prevalence among pediatric patients with AD. The overall pooled prevalence of ASD was 0.81% (95% CI, 0.42%–1.55%) (12, 16, 28, 36, 37, 42), with a 95% prediction interval of 0.07%–8.70% (Supplementary Figure S7). Considerable between-study heterogeneity was observed (*I*^2^ = 99.79%, *τ*^2^ = 0.67, *P* < 0.001). In the exploratory subgroup analyses (Table 2) by geographical region, the pooled prevalence was 0.46% (95% CI, 0.24%–0.87%) across three Asian studies and 1.99% (95% CI, 1.67%–2.36%) across two North American studies (Supplementary Figure S8). The single European study reported a prevalence of 0.71% (95% CI, 0.69%–0.74%) (42). For different study types, the pooled prevalence of ASD was 0.65% (95% CI, 0.52%–0.80%) in cohort studies and 0.99% (95% CI, 0.24%–3.93%) in cross-sectional studies (Supplementary Figure S9). The pooled prevalence was 0.51% (95% CI, 0.31%–0.85%) in studies using ICD or diagnostic codes and 1.99% (95% CI, 1.67%–2.36%) in studies using parental or caregiver reports. All six included studies were population-based or registry-based. Although significant subgroup differences were identified according to geographical region and ASD assessment method (both *P* for subgroup differences <0.001), these results should be interpreted with caution because each subgroup contained few studies and substantial heterogeneity persisted.

#### Learning disability and intellectual disability

All three included studies on LD were cross-sectional and conducted in the US, and the pooled prevalence was 11.34% [95% CI, 7.41%–16.98% (44–46), *I*^2^ = 94.06%, *τ*^2^ = 0.16, *P* < 0.001, Supplementary Figure S11]. Few studies have specifically quantified the prevalence of ID among AD children. Huang et al. (45) reported that the prevalence of ID was approximately 3.05%, while Jackson-Cowan et al. (47) found a developmental delay rate of 6.96%.

#### Other neurodevelopmental outcomes

Six studies investigated conduct disorder (CD). The pooled prevalence of CD in pediatric patients with AD was 2.76% [95% CI, 0.67%–10.67% (14–16, 28, 30, 39), *I*^2^ = 99.49%, *τ*^2^ = 3.18, *P* < 0.001, 95% PI, 0.01–85.67; Supplementary Figure S12]. Three studies explored peer problems and found a pooled prevalence of 5.98% [95% CI, 2.71%–12.68% (14, 15, 39); *I*^2^ = 95.14%, *τ*^2^ = 0.50, *P* < 0.001; Supplementary Figure S13]. Evidence for oppositional defiant disorder (ODD) was limited to two studies, which reported prevalence estimates of 5.6% (31) and 21.3% (30), respectively. Jackson-Cowan et al. (47) and Ma et al. (46) reported the prevalence of self-reported memory impairment as 0.87% and 11.1%, respectively. Only one cross-sectional study examined 80 children aged 3–5 years in Turkey and reported the prevalence of TD among AD patients, which was 6.3% (30).

#### Sensitivity analyses and publication bias

Leave-one-out analyses showed limited influence of individual studies on the pooled prevalence estimates for ADHD and ASD (Supplementary Figures S14, S15). In contrast, the CD estimate was sensitive to the exclusion of Ahn et al. (28) (Supplementary Figure S16). For ADHD prevalence, visual inspection of the funnel plot suggested asymmetry (Supplementary Figure S17), and Egger's regression test indicated potential small-study effects (*P* = 0.007). Trim-and-fill analysis imputed three studies and reduced the pooled prevalence from 6.01% to 4.97%, indicating modest attenuation of the estimate (Supplementary Figure S18). Given the substantial between-study heterogeneity, these findings were interpreted cautiously.

### Association between atopic dermatitis and neurodevelopmental outcomes

#### Attention-deficit/hyperactivity disorder

In our meta-analysis, 18 studies reporting ORs yielded a pooled OR of 1.34 (95% CI, 1.21–1.50, *I*^2^ = 88.57%, *τ*^2^ = 0.03, *P* < 0.001 12, 15, 16, 18, 29, 30, 34, 35, 39, 40, 48–55), with a 95% prediction interval of 0.90–2.01 (Supplementary Figure S19). Among four studies reporting HRs, the pooled estimate was directionally similar but less precise (HR = 1.54, 95% CI, 0.97–2.44, *I*^2^ = 99.58%, *τ*^2^ = 0.22, *P* < 0.001, Supplementary Figure S20) (36, 37, 41, 42). Genuneit et al. (33) separately reported an RR of 5.17 (95% CI, 2.18–12.28). In our exploratory age subgroup analysis, the pooled association was higher among children aged 6–18 years (OR = 2.00, 95% CI, 1.63–2.47) than among those aged <6 years (OR = 1.33, 95% CI, 0.97–1.82), with evidence of a between-subgroup difference (*P* for subgroup difference = 0.03; Supplementary Figure S21). Two studies investigated the impact of age at onset of AD on the association with ADHD. Due to differences in definitions of age at AD onset, quantitative synthesis was not performed. Genuneit et al. (33) reported that early-onset eczema (0–4 years) was strongly associated with early ADHD (RR = 5.17), whereas the association with late ADHD was not statistically significant. Liao et al. (37) observed that AD diagnosed before 2 years was associated with subsequent ADHD diagnosis (HR = 1.16), with greater associations observed among children with earlier onset. The definitions of AD severity varied substantially across studies. Strom et al. (17) and Yaghmaie et al. (16), both based on overlapping NSCH populations and using parent-reported AD severity, showed stronger associations with ADHD at higher levels of AD. Kuniyoshi et al. (52) assessed AD severity using the International Study of Asthma and Allergies in Childhood (ISAAC) symptom questionnaire and also found that severe AD was associated with ADHD (OR = 2.48, 95% CI 1.76–3.52). Wan et al. (42) and Vittrup et al. (41) classified AD severity based on prescription data. Wan et al. reported no significant associations between AD severity and ADHD, whereas Vittrup et al. observed significant associations for moderate AD (HR = 1.84, 95% CI, 1.42–2.38) and severe AD (HR = 3.23, 95% CI, 1.77–5.90). In exploratory analyses of potentially associated factors, sleep disturbances were associated with higher odds of ADHD (OR = 2.15, 95% CI, 1.44–3.20, Supplementary Figure S22) (17, 18, 35, 39). No significant association was observed between obesity and ADHD (OR = 1.43, 95% CI, 0.90–2.28, Supplementary Figure S23) (17, 49). Sex- and race-related findings were reported in three overlapping US survey analyses and were therefore not quantitatively pooled (12, 17, 45). Hou and Silverberg and Strom et al. showed broadly similar patterns, with lower ADHD occurrence among girls and non-White children. In contrast, Huang et al., which was restricted to children with low birth weight, reported stronger associations among girls and non-White children. Other characteristics, including allergic comorbidities, lower educational attainment, lower household income, and parental history of smoking, were also mentioned as being associated with ADHD, as reported by Hou and Silverberg (12) and Strom et al. (17).

#### Autism spectrum disorder

Two studies reporting ORs showed a positive association between AD and ASD (OR = 2.11, 95% CI, 1.07–4.16, *I*^2^ = 91.73%, *τ*^2^ = 0.22, *P* < 0.001, Supplementary Figure S24) (12, 16). The combined HRs from three studies showed a comparable direction of association, although with lower precision (HR = 2.09, 95% CI, 0.54–8.19, *I*^2^ = 99.89%, *τ*^2^ = 1.42, *P* < 0.001, Supplementary Figure S25) (36, 37, 42). For different AD severities, Yaghmaie et al. (16) indicated increasing effect estimates across severity categories, with mild (OR = 1.78, 95% CI, 1.14–2.77), moderate (OR = 3.25, 95% CI, 1.79–5.90), and severe AD (OR = 7.41, 95% CI, 3.82–14.36). Wan et al. (42) found that only moderate AD had a significant association with ASD diagnosis (HR = 1.25, 95% CI, 1.10–1.41).

#### Learning disability and intellectual disability

The pooled analysis of three studies showed that AD was associated with higher odds of learning disability [OR = 1.71, 95% CI, 1.41–2.06 (44–46), *I*^2^ = 0.00%, *τ*^2^ = 0.00, *P* = 0.96, Supplementary Figure S26]. Among them, some studies have pointed out that this relationship may be dose-dependent on the severity of AD (11, 44). AD was also positively associated with intellectual disability (OR = 1.39, 95% CI, 1.33–1.45, *I*^2^ = 0.00%, *τ*^2^ = 0.00, *P* = 0.98, Supplementary Figure S27). Furthermore, most existing research has not identified a meaningful association between AD and general cognitive or academic measures in children (45, 56–58), though a small number of studies have suggested that moderate to severe AD may impair cognition and is associated with delayed educational attainment (58, 59).

#### Other neurodevelopmental outcomes

In our included studies, only one study reported the association between TD and AD children (OR = 1.34, 95% CI, 1.21–1.47) (50). Prior meta-analyses have shown that when TD is considered as the exposure group, the association between TD and AD was not significant (60). Compared with non-AD children, those with AD had higher odds of CD (OR = 1.35, 95% CI, 1.16–1.58, *I*^2^ = 74.03%, *τ*^2^ = 0.02, *P* = 0.01, 95% PI, 0.84–2.19, Supplementary Figure S28) (14–16, 39, 48, 52). No significant overall association was observed between AD and peer problems (OR = 1.10, 95% CI, 0.96–1.26, *I*^2^ = 72.04%, *τ*^2^ = 0.02, *P* = 0.03, 95% PI, 0.72–1.67, Supplementary Figure S29) (14, 15, 39, 48, 52, 61). Both mild-to-moderate and severe AD were associated with CD and peer problems, with stronger associations observed among children with severe AD, as reported by Kuniyoshi et al. (52). While Lin et al. (53) found that AD patients had higher odds of exhibiting symptoms of ODD, Catal et al. (30) did not observe significant results.

### Sensitivity analyses and publication bias

Leave-one-out analyses showed that the pooled ORs for ADHD and CD were relatively stable after the sequential omission of individual studies (Supplementary Figure S30, S32). For the HR-based ADHD analysis, the direction of the association remained consistent, but the pooled estimate became statistically significant after excluding Wan et al. (42), a large population-based cohort in which most children had mild AD (Supplementary Figure S31). The peer-problem estimate was sensitive to the exclusion of Ballardini et al. (48), after which the association became statistically significant (Supplementary Figure S33). Ballardini et al. differed from the other included studies in the timing of AD assessment and in the measurement of peer-related outcomes. Leave-one-out sensitivity analyses were not undertaken for the ASD, LD, and ID association analyses due to the limited number of studies. For the ADHD association analysis, no evidence of significant publication bias was detected by funnel plot inspection (Supplementary Figure S34) and Egger's test (*P* = 0.168).

## Discussion

This systematic review and meta-analysis provided a comprehensive synthesis of neurodevelopmental outcomes among children with atopic dermatitis. The pooled prevalence estimates varied considerably across neurodevelopmental outcomes. Substantial heterogeneity was observed across most prevalence analyses, with wide prediction intervals for several outcomes. Compared with children without AD, those with AD had significantly higher odds of ADHD, ASD, LD, ID, and CD. Notably, although the pooled ORs for ADHD and CD were statistically significant, their 95% prediction intervals included the null value, suggesting that the magnitude and presence of these associations may vary across comparable study settings. The pooled prevalence of ADHD was broadly consistent with that reported in a previous meta-analysis (62), and the positive associations of AD with ADHD, ASD, and CD were also consistent with previous studies (63, 64). Beyond these commonly investigated outcomes, our study further examined a broader range of neurodevelopmental problems. Positive associations were identified for LD and ID, whereas no clear overall association was observed for peer problems. These findings broaden the current understanding of neurodevelopmental comorbidities associated with pediatric AD and suggest that neurodevelopmental assessment in children with AD may need to extend beyond ADHD and ASD.

Exploratory subgroup analyses were conducted to examine potential sources of heterogeneity in prevalence estimates. In our regional analysis, we observed a higher prevalence of ADHD and ASD among children with AD in North America, possibly due to ethnic heterogeneity, regional epidemiological trends, and differences in healthcare systems (62). Prior studies have identified North America as having the highest prevalence of mental disorders, which may explain our findings (65). Sample sources were divided into population- and registry-based studies and clinical sample-based studies. The former includes all AD patients within a specific area or a random sample of the population through a census or health registration form. The latter includes participants recruited from treatment centers, hospitals, or community services, likely representing a subset with higher rates of co-occurring conditions. Clinical samples may therefore be more susceptible to referral and selection bias, as children presenting to treatment settings may have a greater disease burden or more co-occurring conditions than those in the general population. Consistent with this possibility, clinical sample-based studies showed higher prevalence estimates than population- or registry-based studies. Reports on the prevalence of other specific neurodevelopmental outcomes in AD are scarce, precluding further stratified analysis. Thus, well-designed longitudinal studies are necessary to further validate these observations.

We found that the association between AD and ADHD appeared stronger among school-aged children, with a statistically significant difference between age subgroups (*P* for subgroup difference = 0.03). Nevertheless, this finding should be considered exploratory and requires confirmation in future studies. The school-age period is a critical phase for neurodevelopment, characterized by biological, cognitive, social, and emotional changes that heighten susceptibility to environmental and psychological stressors (12, 66). In fact, the relationship between AD and mental disorders may be bidirectional and can persist into adulthood (67). Low academic achievement during the school-age period can adversely affect opportunities for further education and future employment. Furthermore, evidence from longitudinal cohort studies suggests that early-life AD onset may be associated with subsequent ADHD diagnosis. These findings indicate that early recognition of neurodevelopmental difficulties in children with AD may be clinically relevant. However, whether systematic screening or targeted supportive interventions improve outcomes remains to be established in prospective studies. Several studies reported larger effect estimates among children with more severe AD, although the definitions of severity varied substantially and these findings were exploratory. Patients with severe AD may experience more intense itching and greater skin barrier impairment. Neuroimmune and inflammatory pathways, including nerve growth factor and cytokine signaling, have been proposed as possible mechanisms linking AD with neurodevelopmental outcomes (43, 68). The pruritus-scratching cycle in AD also disrupts sleep continuity and quality (69). A recent meta-analysis reported a high prevalence of sleep disorders among children with active AD (70). Bao et al. also found that all specific types of sleep disorders more than double the risk of mental health and learning disorders (71). In school-aged children, chronic sleep deprivation and sleep fragmentation can directly or indirectly lead to daytime fatigue, inattention, memory impairment, and poor academic performance (72). Sleep disturbances could impair the prefrontal cortex, which is critical for cognitive functions and emotional regulation (43). Coincidentally, this region is also a focus of AD neuroimaging research (73, 74). The shared brain activation pathways between sleep problems and neurodevelopmental disorders, along with systemic inflammation (e.g., elevated IL-6), may further underlie the association among AD, sleep disorders, and neurocognitive deficits (75, 76). Some studies have reported that children with AD and concurrent atopic comorbidities (e.g., asthma and allergic rhinitis) had higher risk estimates for NDs (12, 17, 36). A recent study indicated that asthma mediates approximately one-sixth of the association between ADHD and AD (77). The underlying mechanisms linking this association involve multiple biological and psychosocial pathways, with various atopic comorbidities potentially exerting compound effects on the development of mental health issues (36). Previous observational studies have reported positive associations of family history of allergic disease and prenatal environmental exposures with neurodevelopmental outcomes (19, 78–80). This implies that offspring may inherit genetic predispositions and share environmental exposures (81). Chronic systemic inflammation during pregnancy has been found to perturb the intrauterine milieu and affect fetal neurodevelopment (82). However, the implications of these observations for prevention remain uncertain and require prospective evaluation. Although our pooled analysis did not identify a significant association between obesity and ADHD among children with AD, previous studies have suggested shared inflammatory pathways between obesity and AD, including chronic low-grade inflammation and immune dysregulation. Obesity-related metabolic and psychosocial factors may also affect behavioral functioning (83–85). These potential mechanisms warrant further investigation.

Emerging observational studies have examined whether effective AD treatment, including biologic therapy, is associated with neurodevelopmental outcomes (86–88). However, this meta-analysis did not evaluate the effects of AD treatment on neurodevelopmental outcomes. Therefore, it cannot be determined from the current evidence whether dupilumab or other therapies modify these outcomes.

This meta-analysis systematically synthesized the prevalence of multiple neurodevelopmental outcomes and their associations with AD in pediatric populations. Most included studies were population-based and involved large samples. For association analyses, adjusted effect estimates were extracted when available, and ORs, RRs, and HRs were analyzed separately. However, some limitations should be noted. First, outcome ascertainment varied substantially across studies. Although diagnostic criteria such as the Diagnostic and Statistical Manual of Mental Disorders (DSM) and ICD are recommended for diagnosing NDs (89), population-based studies often rely on questionnaires as primary research tools. Across the studies included in our analysis, the majority of diagnostic modalities depended on parent or self-reports and questionnaires, which may introduce response bias. Such subjective reporting may lead to reporting or misclassification bias and contribute to variation in prevalence estimates. Parental stress may also exaggerate the perceived severity of the illness (90). Thus, it is imperative to establish more reliable and consistent diagnostic methods to mitigate the impact of varying disease definitions on the longitudinal characterization of AD and ADHD. Second, substantial between-study heterogeneity was observed in several analyses (*I*^2^ > 90%), particularly for prevalence outcomes. Heterogeneity is a particular concern in meta-analyses of observational studies because of differences in study populations, designs, outcome definitions, and control of confounding (91). The wide prediction intervals observed for several outcomes further indicate considerable variation across study settings (25). Therefore, the pooled estimates should be interpreted as average estimates across heterogeneous study settings rather than as universally applicable measures of prevalence or association. Subgroup analyses should also be considered exploratory, particularly when based on few studies or heterogeneous definitions. The observed differences may reflect variations in study design or diagnostic approaches. For example, the stronger association observed between AD and ADHD among school-aged children may partly be influenced by diagnostic timing. Since ADHD is more commonly identified after school entry, younger children may have fewer opportunities for formal assessment, potentially introducing detection bias. Moreover, surveillance bias cannot be completely excluded. Children with AD, particularly those with more severe disease, may have more frequent healthcare visits and greater opportunities for neurodevelopmental assessments. This may potentially increase the likelihood of ND detection compared with children without AD. Therefore, the observed associations may partly reflect differences in healthcare utilization rather than solely underlying biological mechanisms. Third, the included evidence was observational, which does not imply causality but only association. In many studies, particularly cross-sectional analyses, the temporal sequence between AD and neurodevelopmental outcomes could not be determined. Although adjusted effect estimates were used for the association analyses, the covariates included in the original multivariable models varied across studies, and residual confounding related to differences in covariate adjustment cannot be excluded. Fourth, the available evidence was unevenly distributed across different NDs. While ADHD and ASD have been relatively well studied, other NDs such as LD and ID remain insufficiently studied. Several pooled estimates, particularly those based on a limited number of studies or sensitive to the omission of individual studies, should be interpreted cautiously. Although NOS and AHRQ assessments provide useful information on study-level methodological characteristics, numerical scores alone do not establish the certainty of the underlying evidence. Potential selection bias, outcome misclassification, residual confounding, surveillance/detection bias, differences in covariate adjustment, and incomplete reporting of missing data may still reduce confidence in the findings. Statistical robustness in sensitivity analyses should therefore not be interpreted as high certainty of the underlying evidence. Potential contributing factors, including antihistamine use, socioeconomic status, educational conditions, serum levels, prenatal or maternal exposures, and allergic comorbidities, were not fully assessed due to insufficient data (11, 12, 17, 92). Future studies should evaluate these factors using standardized definitions and longitudinal designs to clarify temporal relationships and better inform potential preventive strategies.

## Conclusion

This systematic review and meta-analysis provided a comprehensive synthesis of neurodevelopmental outcomes in pediatric patients with AD. Pooled prevalence estimates varied across neurodevelopmental outcomes, and AD was associated with higher odds of ADHD, ASD, LD, ID, and CD, whereas no clear overall association was observed for peer problems. HR-based estimates for ADHD and ASD were directionally similar but less precise. Substantial heterogeneity, potential detection bias, and the observational nature of the evidence limit generalizability and causal interpretation. Further well-designed longitudinal studies using standardized definitions and outcome assessments are needed to clarify temporal relationships and the consistency of these associations.

## Data Availability

The original contributions presented in the study are included in the article/Supplementary Material, further inquiries can be directed to the corresponding authors.
