# Supplementary material for: Neurodevelopmental disorders in pediatric patients with atopic dermatitis: a systematic review and meta-analysis
Source: Front Med (Lausanne). 2026 Oct 29;13:1975921. doi: 10.3389/fmed.2026.1975921 (PMC13754735; doi:10.3389/fmed.2026.1975921)
Supplement: Supplementary file 1 [file Datasheet1.pdf]

Table S1. Search strategy used for the electronic databases

Database	Query	Results
**PubMed**	(((((((((("Dermatitis, Atopic"[Mesh]) OR (Atopic Dermatitis[Title/Abstract])) OR (Atopic Eczema[Title/Abstract])) OR (Infantile Eczema[Title/Abstract])) OR (Infantile Eczema[Title/Abstract])) OR (Atopic Neurodermatitis[Title/Abstract])) OR (Disseminated Neurodermatitis[Title/Abstract])) OR (childhood eczema[Title/Abstract])) OR (Besnier's prurigo[Title/Abstract])) OR (Besnier's[Title/Abstract])) OR (prurigo[Title/Abstract])) AND (("Neurodevelopmental Disorders"[Mesh] OR "Neurodevelopmental Disorders"[Title/Abstract] OR "Attention Deficit and Disruptive Behavior Disorders"[Mesh] OR "Attention Deficit and Disruptive Behavior Disorders"[Title/Abstract] OR "Attention Deficit Disorder with Hyperactivity"[Mesh] OR "Attention Deficit Disorder with Hyperactivity"[Title/Abstract] OR "Attention Deficit Hyperactivity Disorder"[Title/Abstract] OR "Hyperkinetic Syndrome"[Title/Abstract] OR "Hyperkinetic Disorder"[Title/Abstract] OR "Communication Disorders"[Mesh] OR "Oppositional Defiant Disorder"[Title/Abstract] OR "Conduct Disorder"[Mesh] OR "Conduct Disorder"[Title/Abstract] OR "Autism Spectrum Disorder"[Mesh] OR "Autism Spectrum Disorder"[Title/Abstract] OR "Autistic Disorder"[Mesh] OR "Autistic Disorder"[Title/Abstract] OR "Child Behavior Disorders"[Mesh] OR "Child Behavior Disorders"[Title/Abstract] OR "Learning Disabilities"[Mesh] OR "Learning Disabilities"[Title/Abstract] OR "Learning Disorder"[Title/Abstract] OR "Tic Disorders"[Mesh] OR "Tic Disorders"[Title/Abstract] OR "Tourette Syndrome"[Mesh] OR "Tourette Syndrome"[Title/Abstract] OR "Developmental Disabilities"[Mesh] OR "Developmental Disability"[Title/Abstract] OR "Developmental Disabilities"[Title/Abstract] OR "Intellectual Disability"[Mesh] OR "Intellectual Disability"[Title/Abstract] OR "Motor Skills Disorders"[Mesh] OR "Motor Disorders"[Title/Abstract])	297
**WOS**	TS=(Neurodevelopmental Disorders OR Learning Disabilities OR Learning Disorder OR Attention Deficit and Disruptive Behavior Disorders OR Conduct Disorder OR Intellectual Disability OR Autistic Disorder OR Autism Spectrum Disorder OR Attention Deficit Disorder with Hyperactivity OR ADHD OR Attention Deficit Hyperactivity Disorder OR Hyperkinetic Syndrome OR Attention Deficit Hyperactivity Disorders OR Minimal Brain Dysfunction OR Oppositional Defiant Disorder OR Child Behavior Disorders OR Tic Disorders OR Tourette Syndrome OR Developmental Disability OR Motor Disorders) AND TS=(Atopic Dermatitis OR Atopic Neurodermatitis OR Disseminated Neurodermatitis OR Atopic Eczema OR Infantile Eczema OR Childhood Eczema OR Bernier’s prurigo)	818
**PsycINFO**	TX (Atopic Dermatitis OR Atopic Neurodermatitis OR Disseminated Neurodermatitis OR Atopic Eczema OR Infantile Eczema OR Childhood Eczema OR Besnier’s prurigo) AND TX (Neurodevelopmental Disorders OR Learning Disabilities OR Learning Disorder OR Attention Deficit and Disruptive Behavior Disorders OR Conduct Disorder OR Intellectual Disability OR Autistic Disorder OR Autism Spectrum Disorder OR Attention Deficit Disorder with Hyperactivity OR ADHD OR Attention Deficit Hyperactivity Disorder OR Hyperkinetic Syndrome OR Attention Deficit Hyperactivity Disorders OR Minimal Brain Dysfunction OR Oppositional Defiant Disorder OR Child Behavior Disorders OR Tic Disorders OR Tourette Syndrome OR Developmental Disability OR Motor Disorders)	252
**Embase**	#3 #2 AND #1<br>#2 'attention deficit hyperactivity disorder'/exp OR 'autism'/exp OR 'learning disorder'/exp OR 'learning disorder'/exp OR 'intellectual impairment'/exp OR 'tic'/exp OR 'neurodevelopmental disorders':ti,ab,kw OR 'learning disorder':ti,ab,kw OR 'attention deficit hyperactivity disorder':ti,ab,kw OR 'autism':ti,ab,kw OR 'intellectual impairment':ti,ab,kw OR 'conduct disorder':ti,ab,kw OR 'Oppositional Defiant Disorder':ti,ab,kw OR 'Child Behavior Disorders':ti,ab,kw OR 'Tic Disorders':ti,ab,kw OR 'Developmental Disabilities':ti,ab,kw	1,128

	#1 'atopic dermatitis':ti,ab,kw OR 'atopic eczema':ti,ab,kw OR 'infantile eczema':ti,ab,kw OR 'childhood eczema':ti,ab,kw OR 'atopic dermatitis'/exp	
**Cochrane Library**	#1 MeSH descriptor: [Dermatitis, Atopic] explode all trees<br>#2 ("atopic dermatitis"):ti,ab,kw<br>#3 (Atopic Neurodermatitis):ti,ab,kw<br>#4 ("disseminated neurodermatitis"):ti,ab,kw<br>#5 ("atopic eczema"):ti,ab,kw<br>#6 ("infantile eczema"):ti,ab,kw<br>#7 (Childhood Eczema):ti,ab,kw<br>#8 (Bernier' prurigo):ti,ab,kw<br>#9 (prurigo):ti,ab,kw<br>#10 #1 OR #2 OR #3 OR #4 OR #5 OR #6 OR #7 OR #8 OR #9<br>#11 MeSH descriptor: [Tic Disorders] explode all trees<br>#12 ("TICS"):ti,ab,kw<br>#13 ("Tourette syndrome"):ti,ab,kw<br>#14 MeSH descriptor: [Autism Spectrum Disorder] explode all trees<br>#15 ("autistic disorder"):ti,ab,kw<br>#16 ("autistic"):ti,ab,kw<br>#17 MeSH descriptor: [Attention Deficit Disorder with Hyperactivity] explode all trees<br>#18 ("attention deficit disorder"):ti,ab,kw<br>#19 (ADHD):ti,ab,kw<br>#20 ("attention deficit hyperactivity disorder"):ti,ab,kw<br>#21 (Neurodevelopmental Disorders):ti,ab,kw<br>#22 MeSH descriptor: [Neurodevelopmental Disorders] explode all trees<br>#23 ("learning disabilities"):ti,ab,kw<br>#24 MeSH descriptor: [Learning Disabilities] explode all trees<br>#25 MeSH descriptor: [Motor Disorders] explode all trees<br>#26 ("motor disorder"):ti,ab,kw<br>#27 ("intellectual disability"):ti,ab,kw<br>#28 MeSH descriptor: [Intellectual Disability] explode all trees<br>#29 ("oppositional defiant disorder"):ti,ab,kw<br>#30 MeSH descriptor: [Oppositional Defiant Disorder] explode all trees<br>#31 #11 OR #12 OR #13 OR #14 OR #15 OR #16 OR #17 OR #18 OR #19 OR #20 OR #21 OR #22 OR #23 OR #24 OR #25 OR #26 OR #27	19

	OR #28 OR #29 OR #30
#32	#31 AND #10

Table S2. Methodological assessment of included studies using the NOS and AHRQ criteria.

2.1 Methodological assessment of case-control studies

Author	Is the case definition adequate	Representativeness of the Cases	Selection of Controls	Definition of Controls	Comparability of Cases and Controls on the Basis of the Design or Analysis	Ascertainment of exposure	Same method of ascertainment for cases and controls	Non-Response rate	Total scores
Horev, 2017	★	★	☆	★	★★	☆	★	★	7

2.2 Methodological assessment of cohort studies

Author	Representativeness of the exposed cohort	Selection of the non-exposed cohort	Ascertainment of exposure	Demonstration that outcome of interest was not present at start of study	Comparability of cohorts on the basis of the design or analysis	Assessment of outcome	Was follow-up long enough for outcomes to occur	Adequacy of follow up of cohorts	Total scores
Ballardini, 2019	★	★	★	☆	★★	★	★	★	8
Genuneit, 2014	★	★	★	★	★★	★	★	★	9
Johansson, 2017	★	★	★	★	★★	★	★	★	9
Kim, 2023	★	★	★	★	★★	★	☆	☆	7
Lee, 2016	★	★	★	★	★★	★	★	★	9
Liao, 2016	★	★	★	★	★★	★	★	★	9
Schmitt, 2010	★	★	★	★	★★	★	★	★	9
Schmitt, 2011	★	★	★	★	★★	★	★	★	9
Shyu, 2012	★	★	★	★	★★	★	☆	☆	7
Vittrup, 2021	★	★	★	★	★★	★	★	★	9
Wan, 2023	★	★	★	★	★★	★	★	★	9

2.3 Methodological assessment of cross-sectional studies

Author	Define the source of informatio n	List inclusion and exclusion criteria for exposed and unexposed subjects or refer to previous publication s	Indicate time period used for identifyin g patients	Indicate whether or not subjects were consecutiv e if not population -based	Indicate if evaluators of subjective components of study were masked to other aspects of the status of the participants	Describe any assessment s undertake n for quality assurance purposes	Explain any patient exclusion s from analysis	Describe how confoundin g was assessed and/or controlled	If applicable , explain how missing data were handled in the analysis	Summar ize patient response rates and complete ness of data collectio n	Clarify what follow-up, if any, was expected and the percentage of patients for which incomplete data or follow-up was obtained	Total score s
Ahn, 2019	Yes	Yes	Yes	Yes	No	Yes	No	Yes	No	Yes	Unclear	7
Augustin, 2015	Yes	No	Yes	Yes	Unclear	Yes	No	No	No	Yes	Unclear	5
Boemanns, 2023	Yes	No	Yes	No	No	Yes	No	Yes	No	Yes	Unclear	5
Catal, 2016	Yes	No	Yes	No	No	Yes	Yes	No	Yes	No	Unclear	5
Cheng, 2021	Yes	No	Yes	Yes	No	Yes	Yes	Yes	Yes	Yes	Unclear	8
Feng, 2020	Yes	Yes	Yes	No	No	Yes	Yes	Yes	No	No	Unclear	6
Fishbein, 2021	Yes	Yes	Yes	Yes	No	Yes	No	Yes	No	Yes	Unclear	7
Hou, 2021	Yes	No	Yes	Yes	No	Yes	Yes	Yes	Yes	Yes	Unclear	8
Hsu, 2019	Yes	Yes	Yes	No	No	Yes	Yes	Yes	No	Yes	Unclear	7
Huang, 2021	Yes	Yes	Yes	Yes	Unclear	Yes	No	Yes	No	Yes	Unclear	7
Huang, 2024	Yes	No	Yes	No	No	Yes	Yes	Yes	Yes	Yes	Unclear	7
Jackson-Cowan, 2021	Yes	No	Yes	Yes	No	Yes	Yes	Yes	No	Yes	Unclear	7
Kuniyoshi, 2018	Yes	No	Yes	Yes	No	Yes	Yes	Yes	No	Yes	Unclear	7
Lin, 2016	Yes	No	Yes	Yes	No	Yes	Yes	Yes	Yes	Yes	Unclear	8
Ma, 2024	Yes	No	Yes	Yes	No	Yes	No	Yes	No	Yes	Unclear	6
Madulara, 2021	Yes	No	No	Yes	No	Yes	Yes	No	Yes	Yes	Unclear	6
Romanos, 2010	Yes	Yes	Yes	No	Unclear	Yes	Yes	Yes	Yes	Yes	Unclear	8
Schmitt, 2009	Yes	Yes	Yes	Yes	Unclear	Yes	No	Yes	No	Yes	Unclear	7

Sockler, 2024	Yes	No	Yes	Yes	No	Yes	No	Yes	Yes	Yes	Yes	8
Strom, 2016	Yes	No	Yes	No	No	Yes	Yes	Yes	Yes	Yes	Unclear	7
Wan, 2020	Yes	Yes	Yes	Yes	No	Yes	No	Yes	No	Yes	Unclear	7
Wan, 2020	Yes	Yes	Yes	Yes	No	Yes	No	Yes	No	Yes	Unclear	7
Wan, 2021	Yes	Yes	Yes	Yes	No	Yes	No	No	No	Yes	Unclear	6
Wong, 2022	Yes	No	No	No	No	Yes	No	Yes	Yes	Yes	Unclear	5
Yaghmaie, 2013	Yes	No	Yes	Yes	No	Yes	No	Yes	No	Yes	Unclear	6
Yang, 2018	Yes	No	Yes	Yes	No	Yes	Yes	Yes	No	Yes	Unclear	7
Yu, 2024	Yes	Yes	Yes	No	No	Yes	No	Yes	No	No	Unclear	5

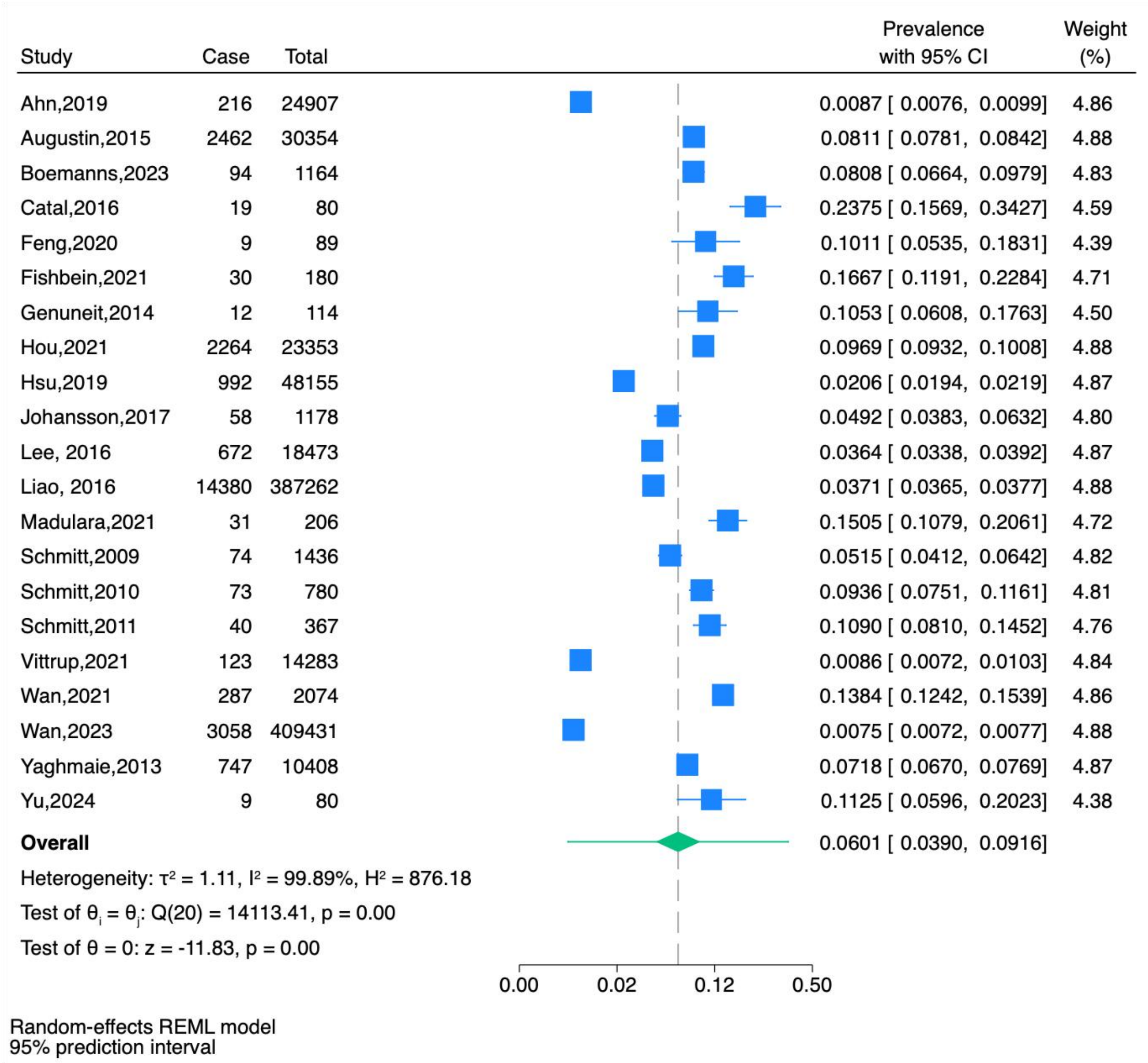

Figure S1. Forest plots of prevalence for ADHD in pediatric patients with AD.

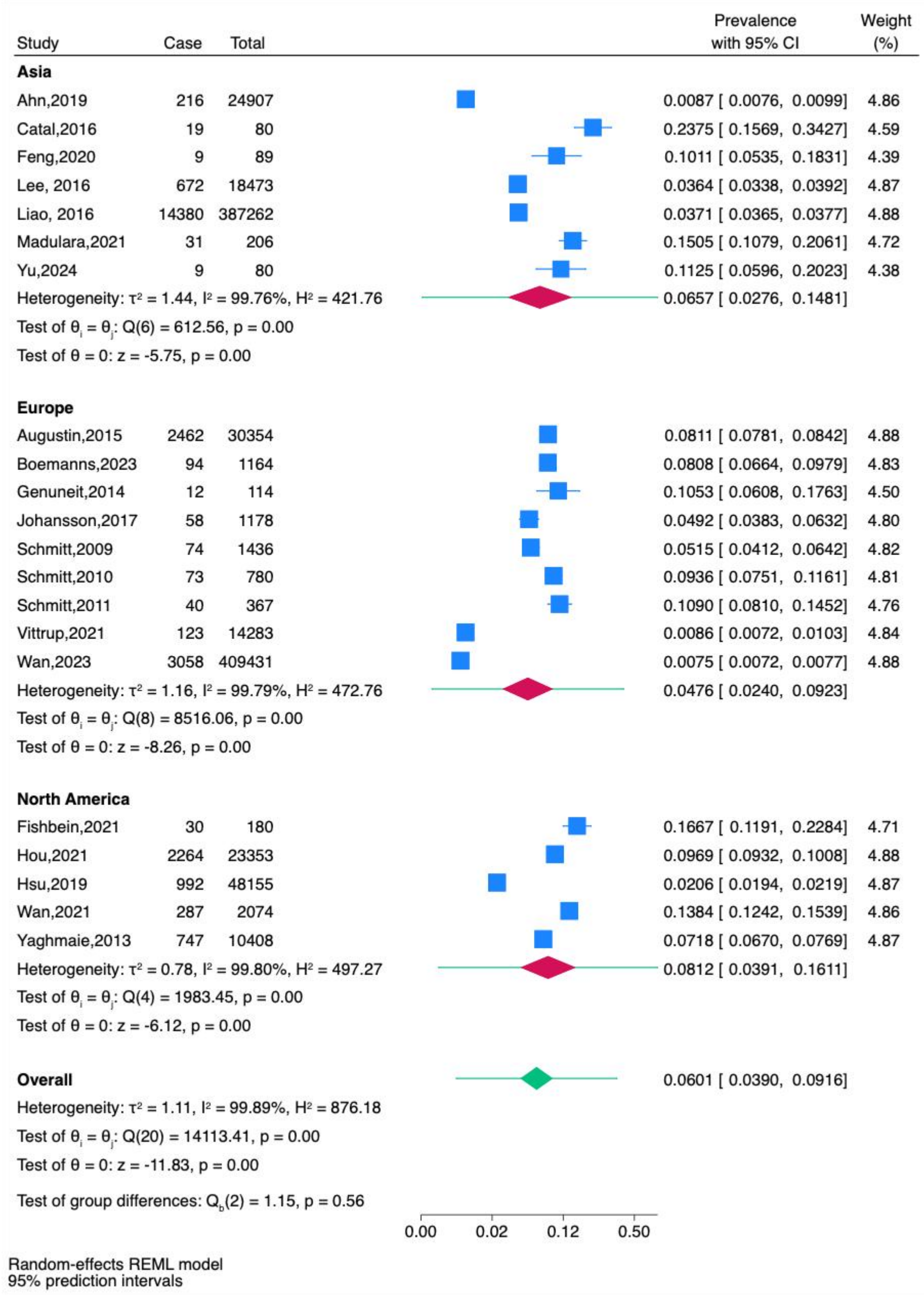

Figure S2. Exploratory subgroup analysis of ADHD prevalence among pediatric patients with AD by geographical region.

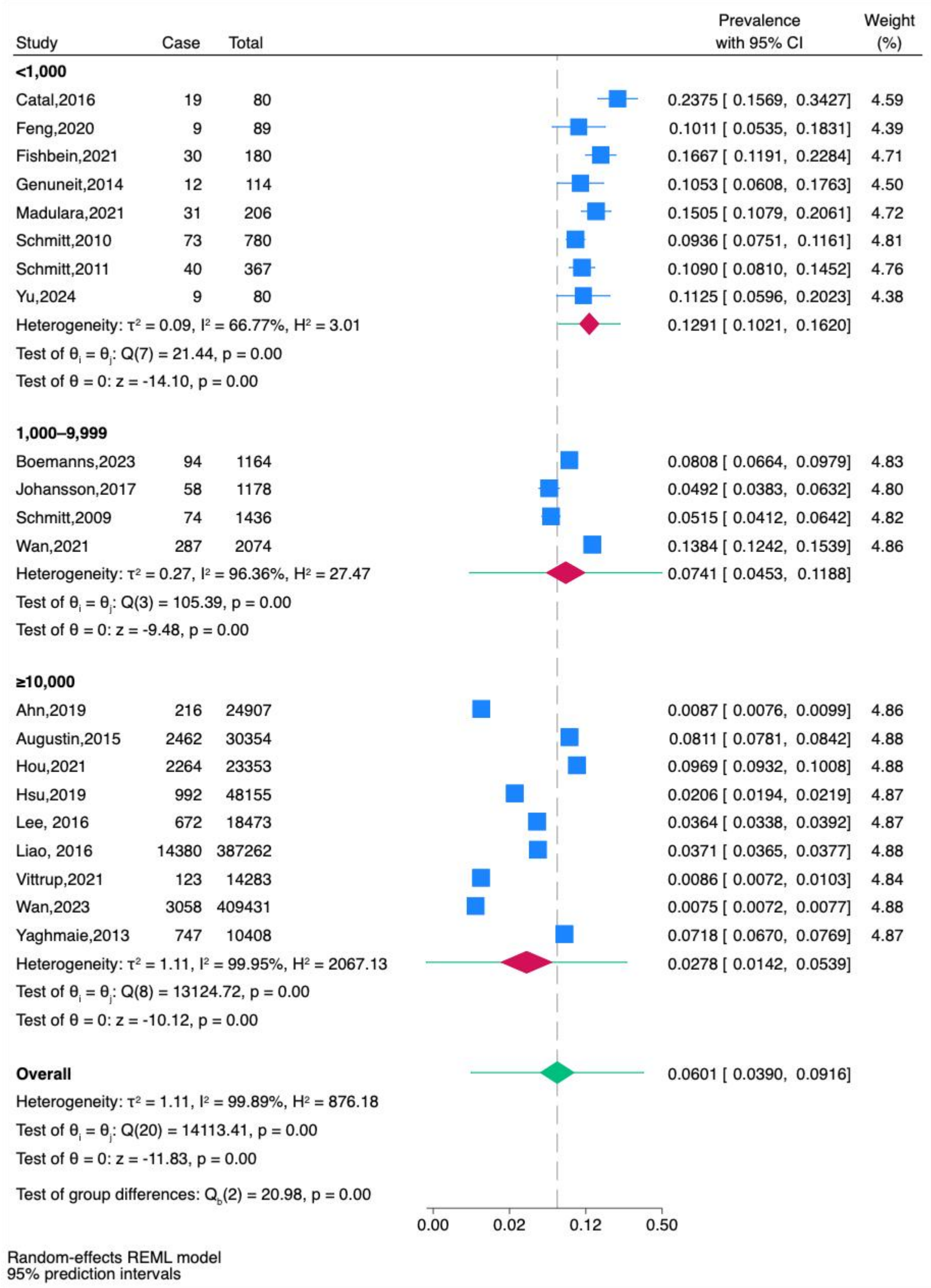

Figure S3. Exploratory subgroup analysis of ADHD prevalence among pediatric patients with AD by sample size.

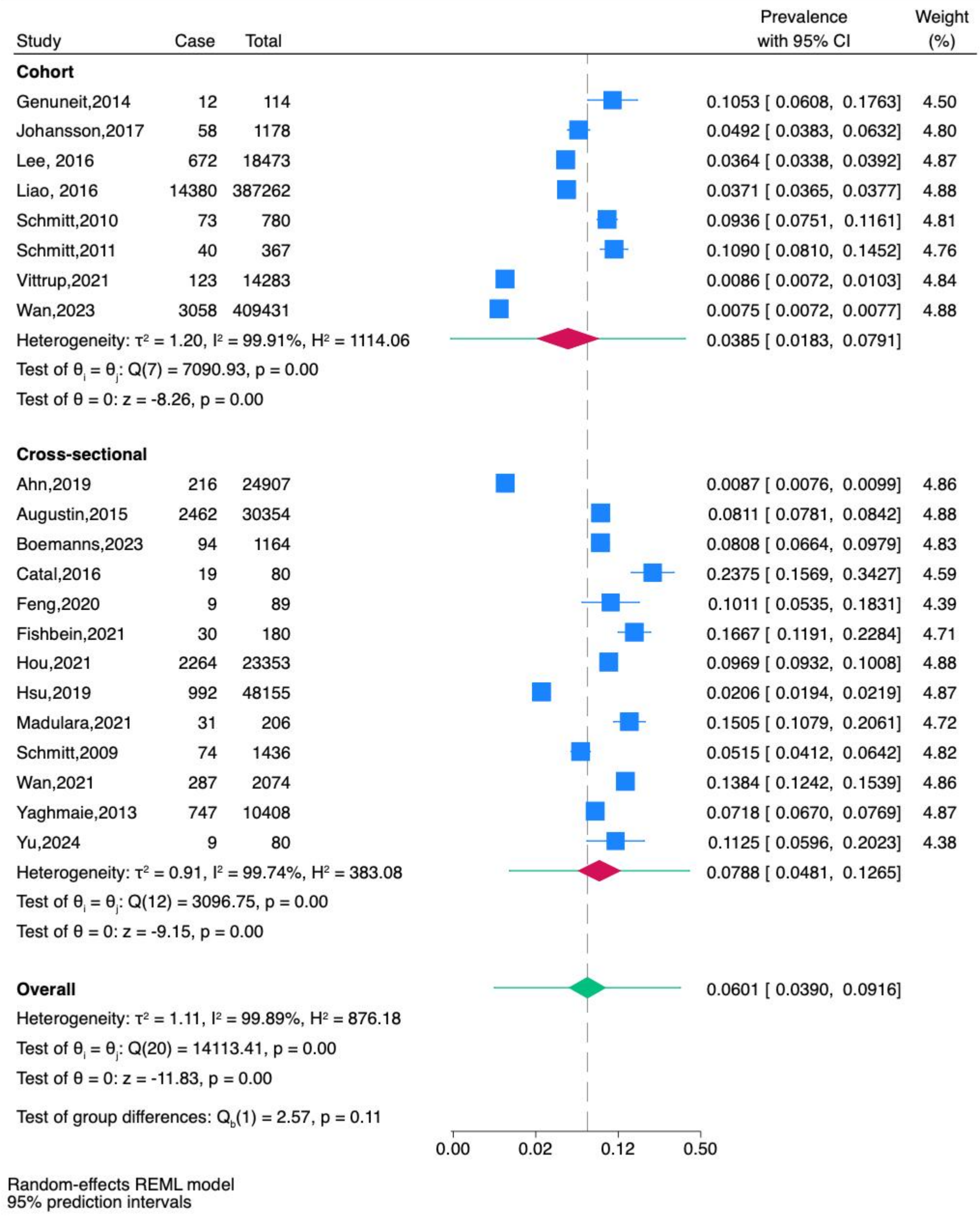

Figure S4. Exploratory subgroup analysis of ADHD prevalence among pediatric patients with AD by study design.

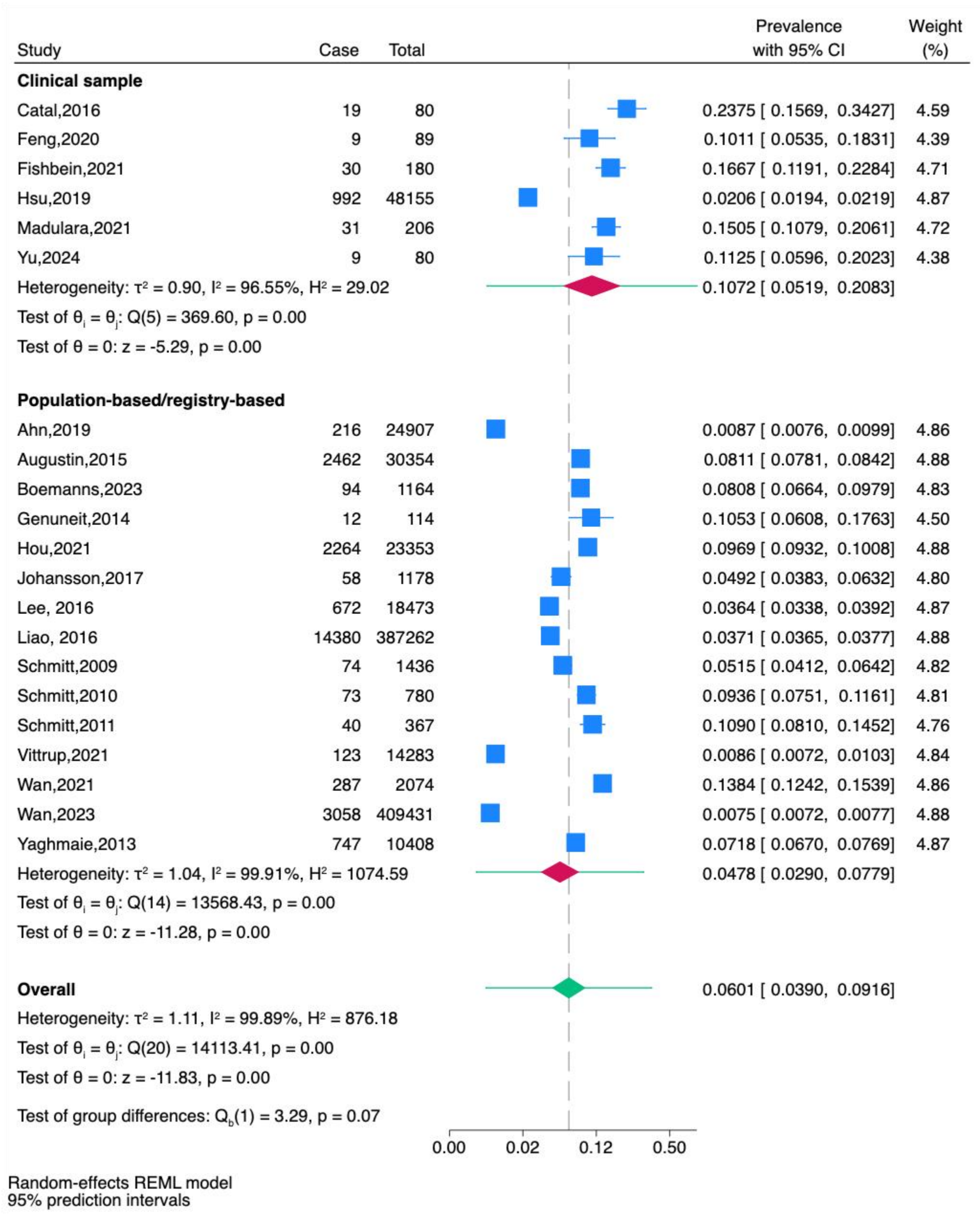

Figure S5. Exploratory subgroup analysis of ADHD prevalence among pediatric patients with AD by source of study population.

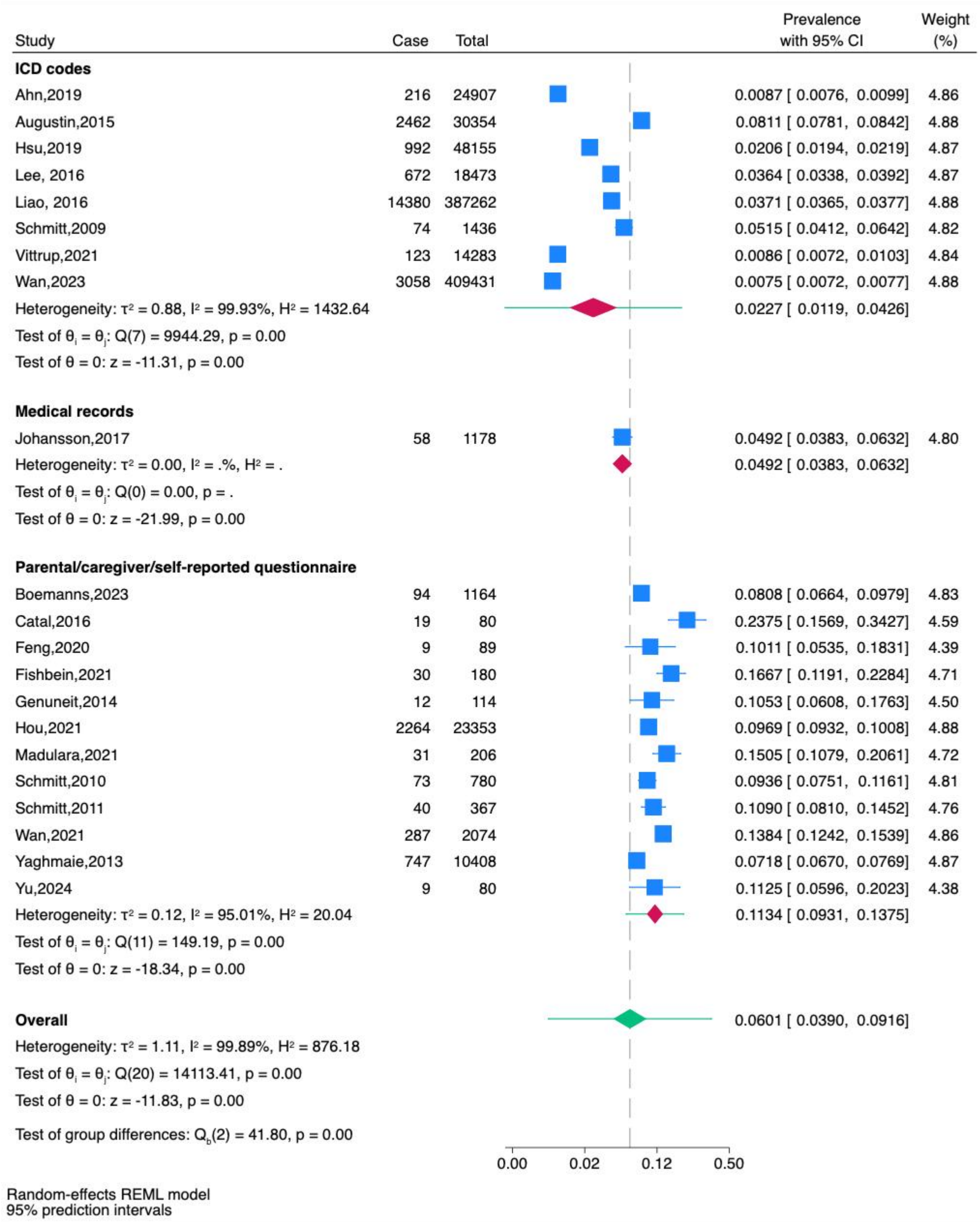

Figure S6. Exploratory subgroup analysis of ADHD prevalence among pediatric patients with AD by ADHD assessment.

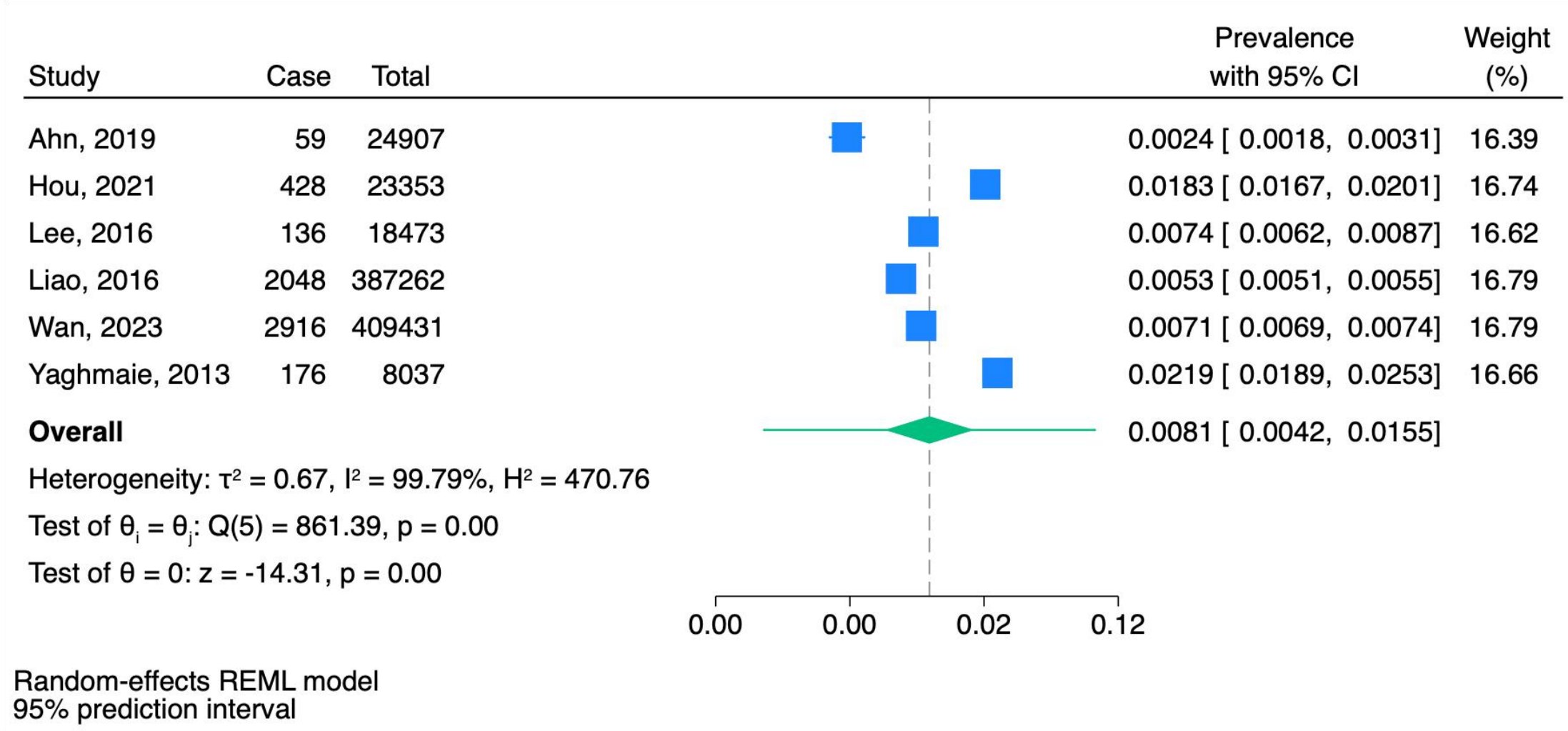

Figure S7. Forest plots of prevalence for ASD in pediatric patients with AD.

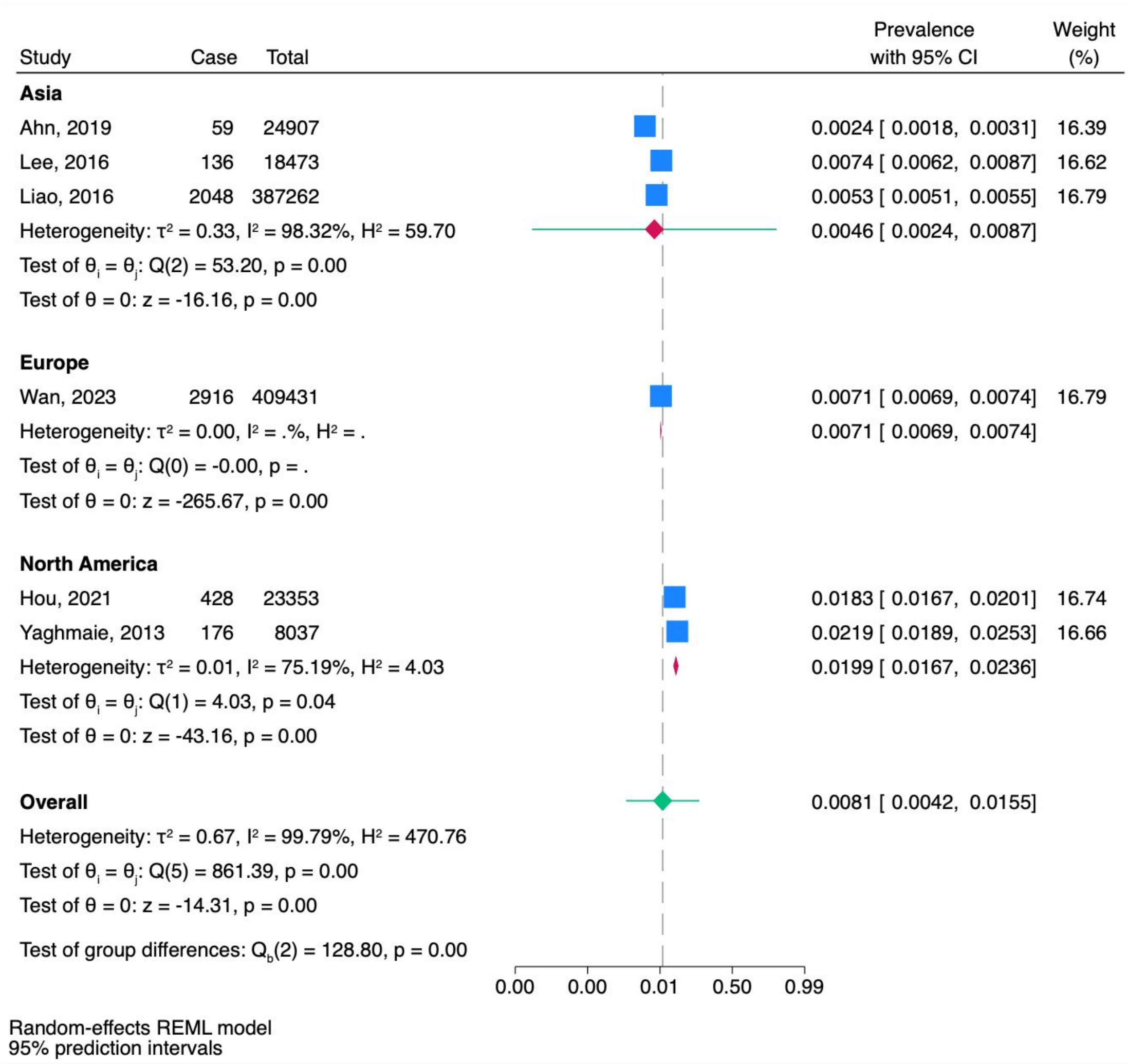

Figure S8. Exploratory subgroup analysis of ASD prevalence in pediatric patients with AD by region.

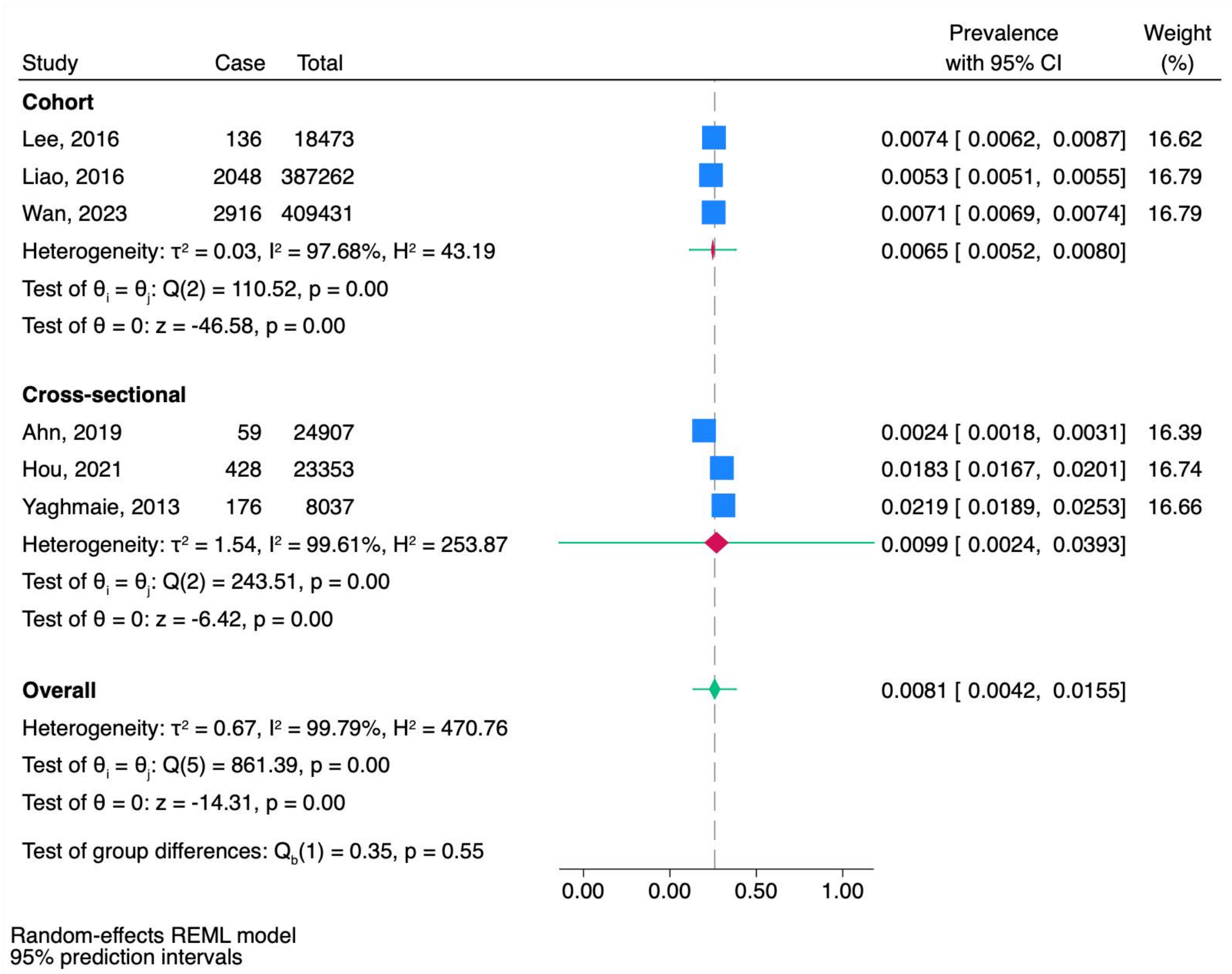

Figure S9. Exploratory subgroup analysis of ASD prevalence in pediatric patients with AD by study design.

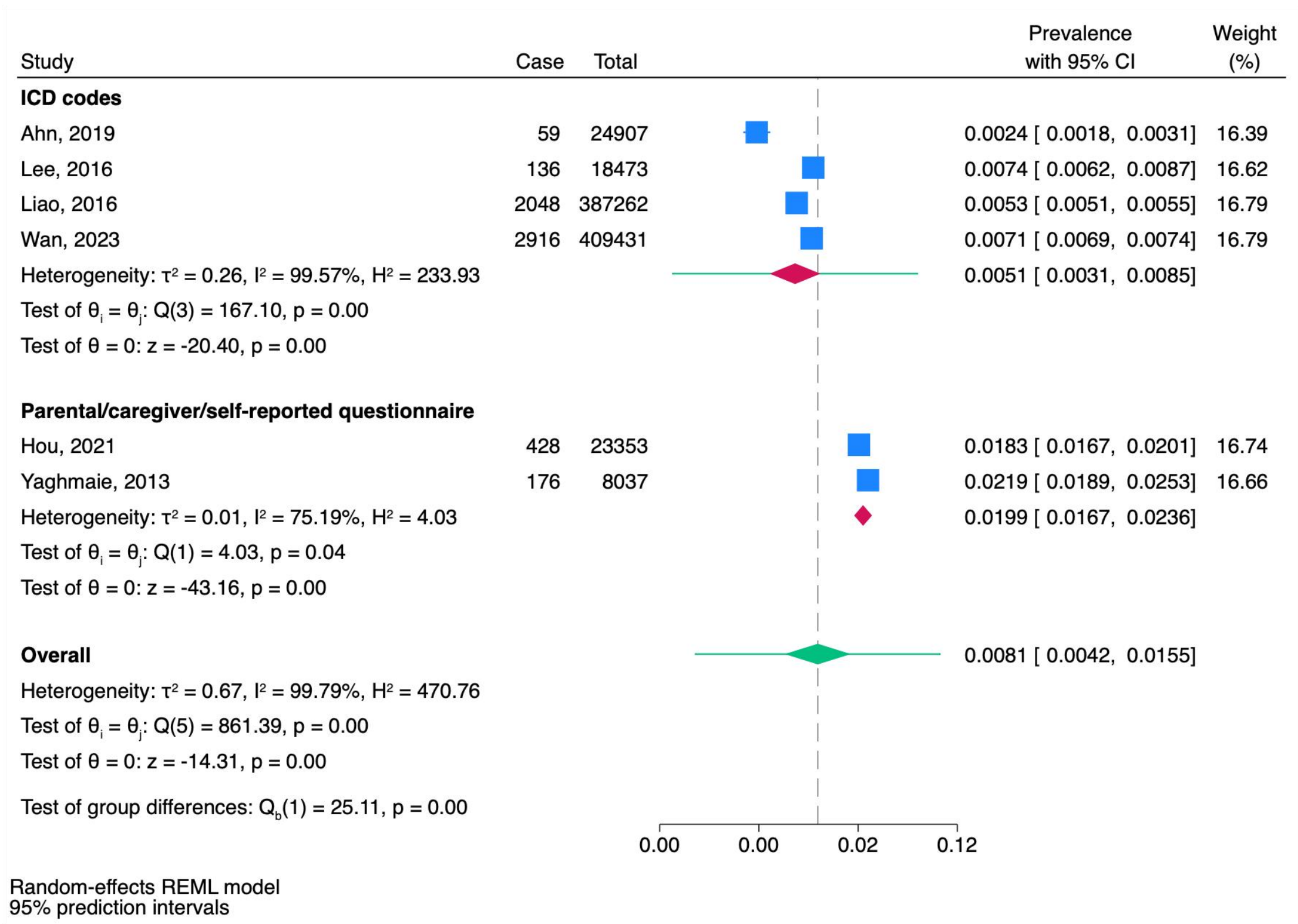

Figure S10. Exploratory subgroup analysis of ASD prevalence among pediatric patients with AD by ASD assessment.

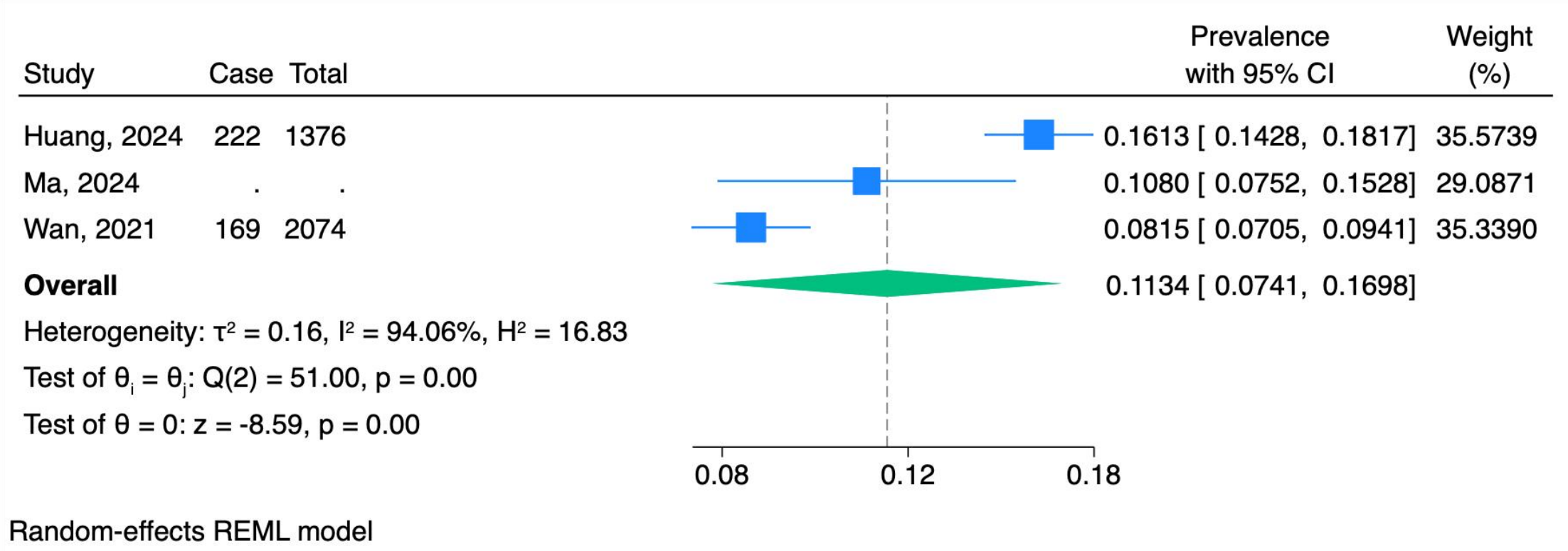

Figure S11. Forest plots of prevalence for learning disability in pediatric patients with AD.

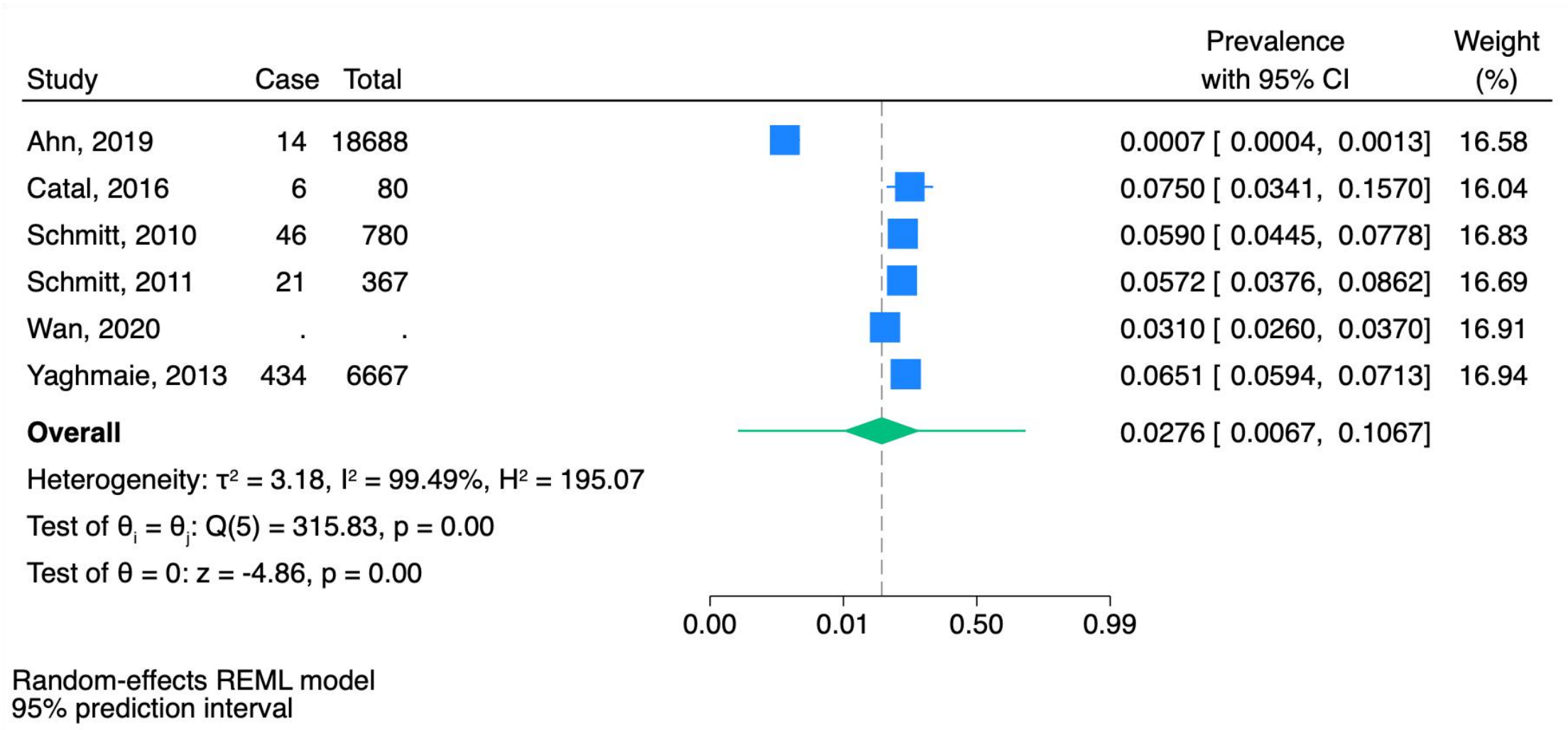

Figure S12. Forest plots of prevalence for conduct disorder in pediatric patients with AD.

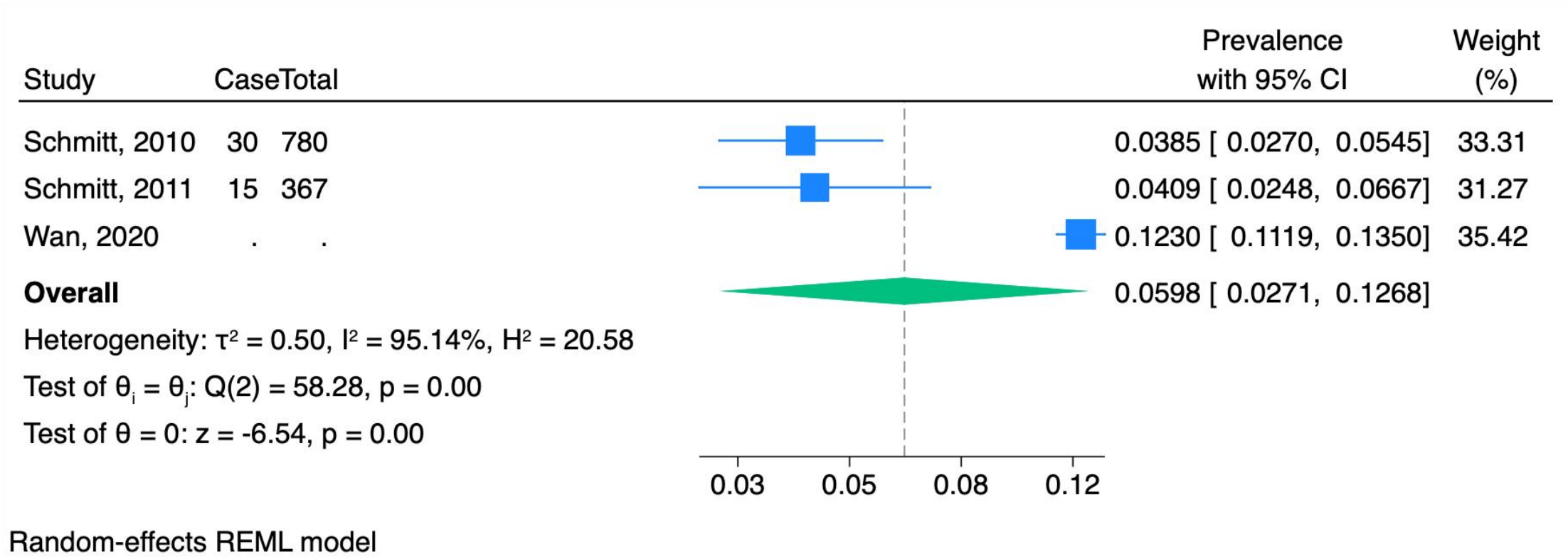

Figure S13. Forest plots of prevalence for peer problems in pediatric patients with AD.

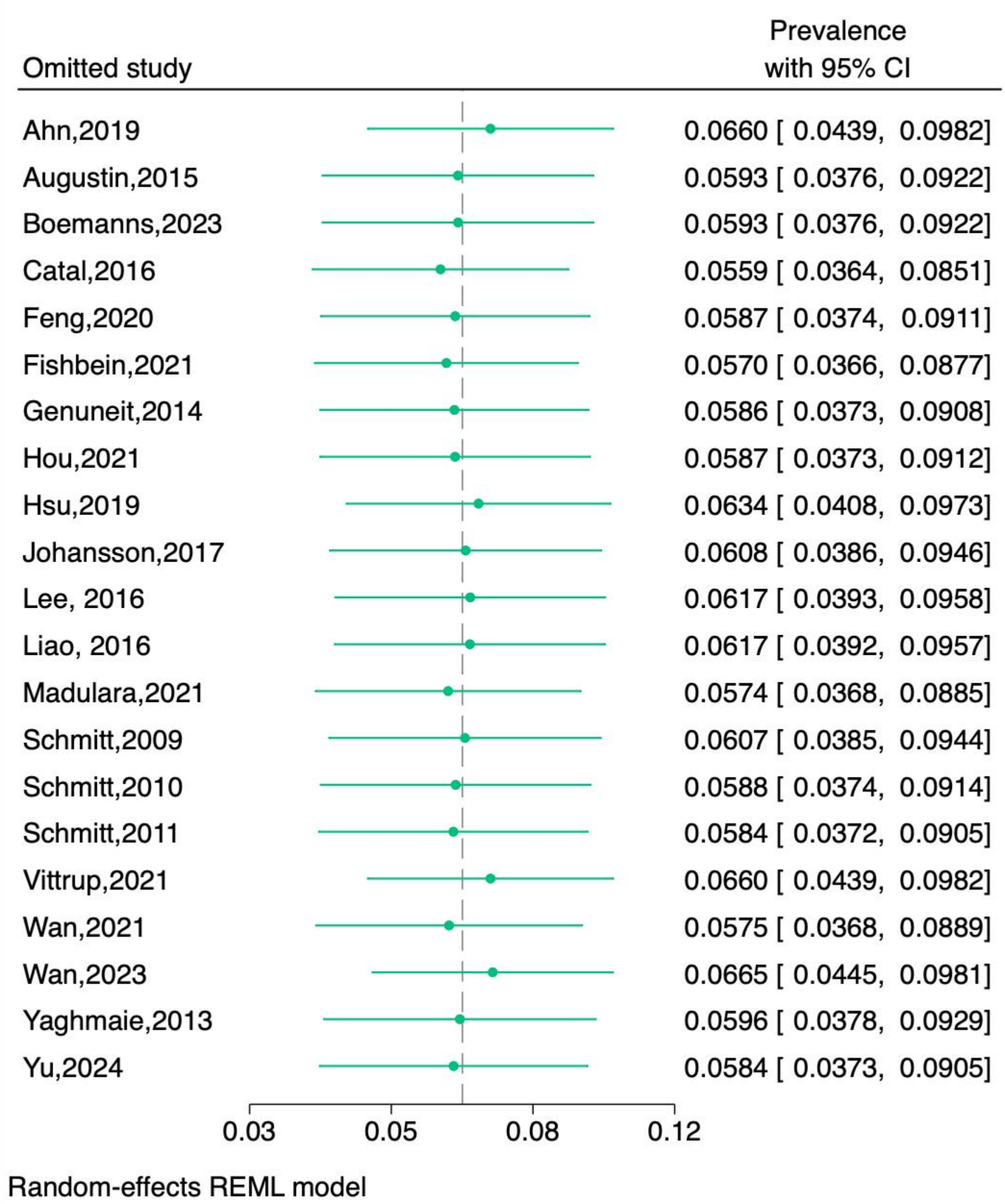

Figure S14. Sensitivity analysis of AD-ADHD prevalence.

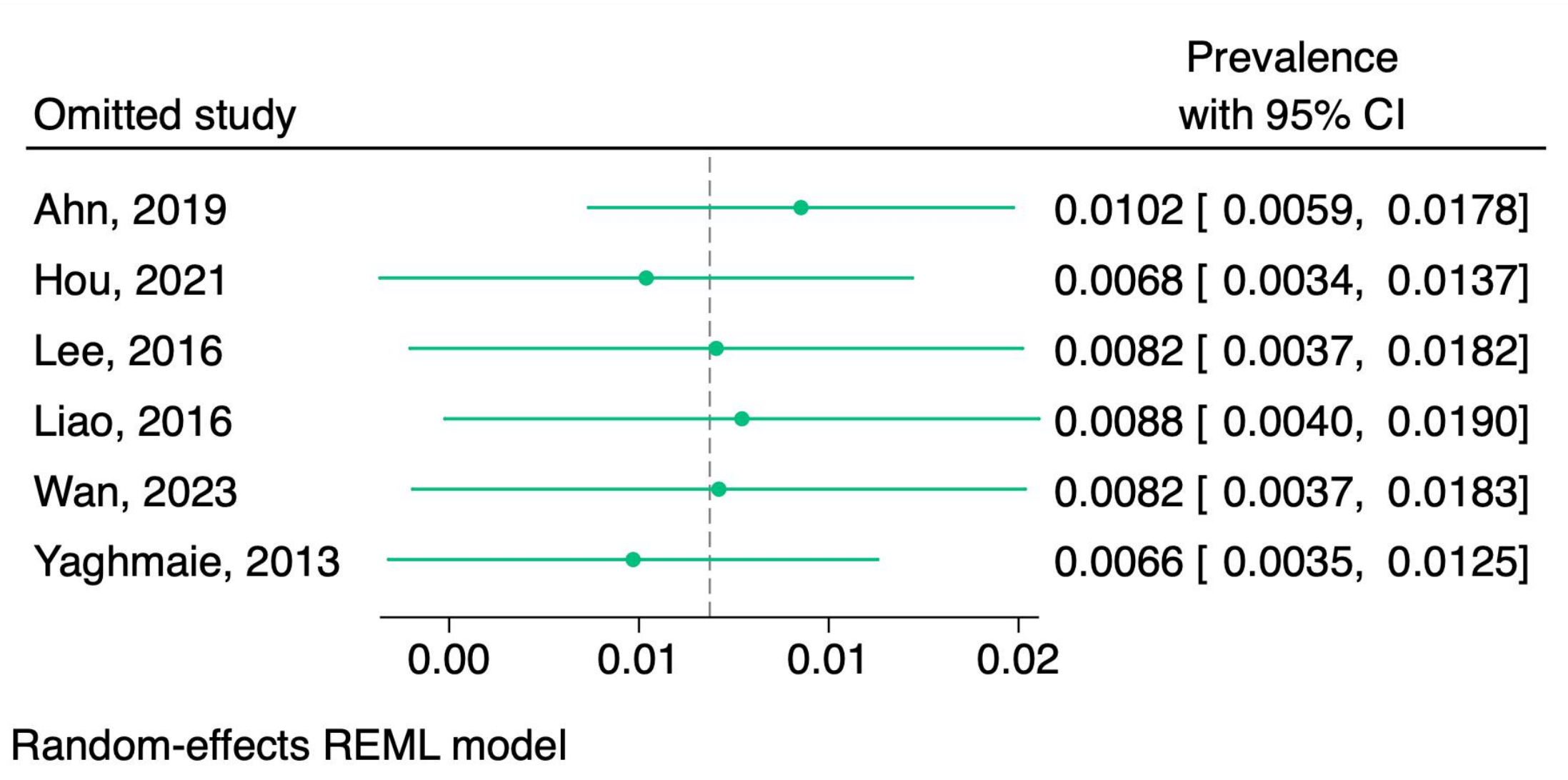

Figure S15. Sensitivity analysis of AD-ASD prevalence.

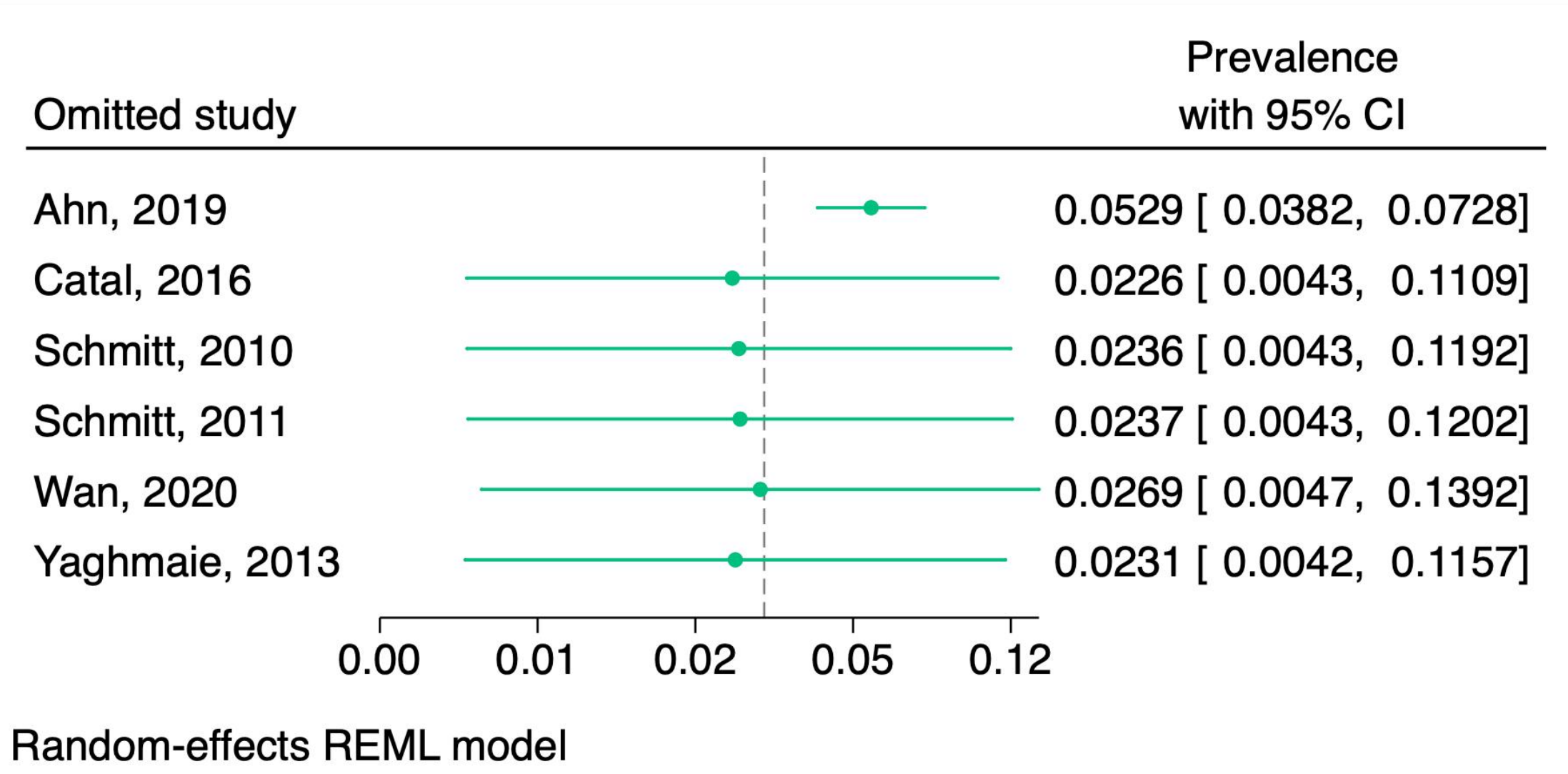

Figure S16. Sensitivity analysis of prevalence for conduct disorder in pediatric patients with AD.

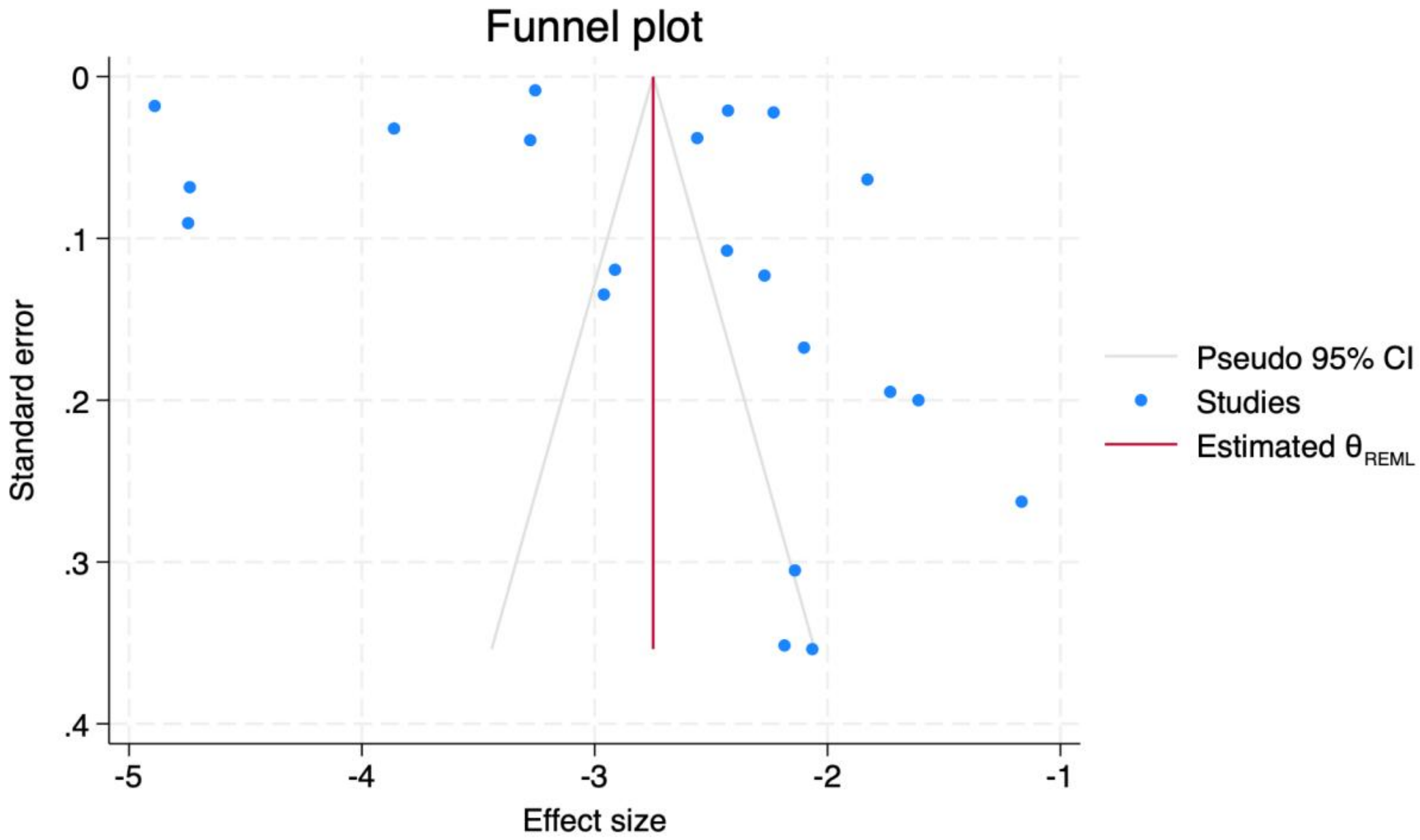

Figure S17. Funnel plot of the prevalence of AD-ADHD.

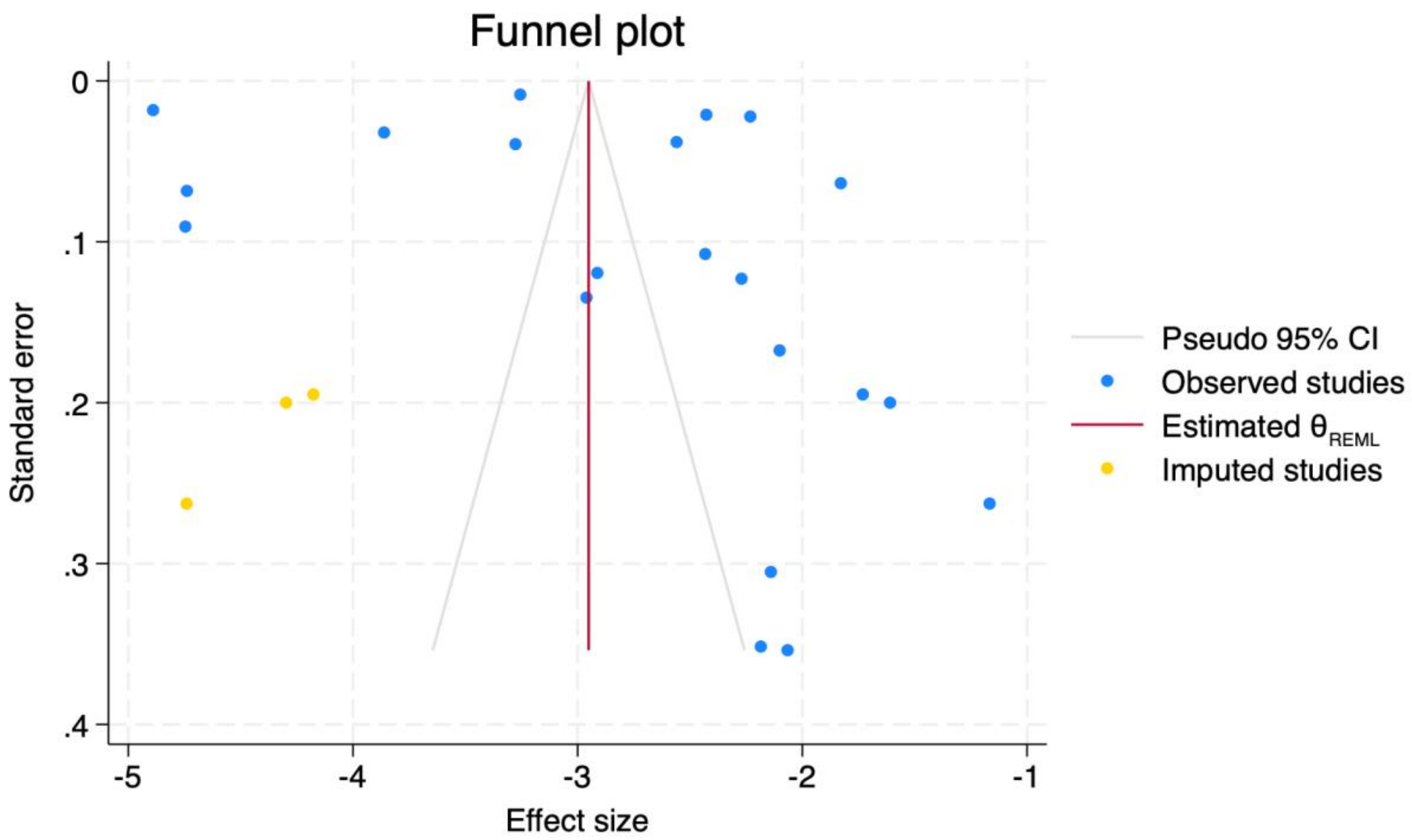

Figure S18. Trim-and-fill analysis for the prevalence of AD-ADHD.

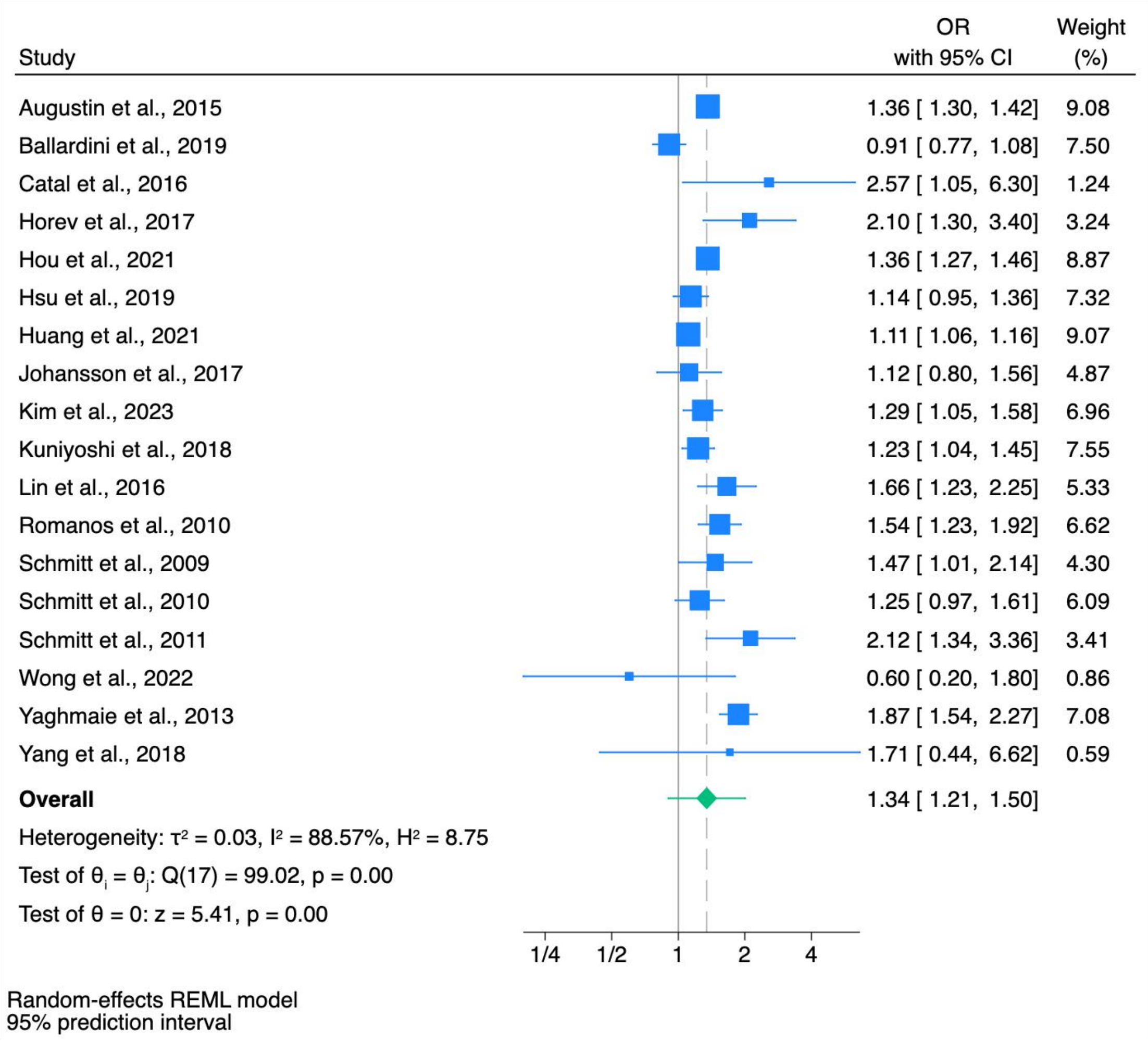

Figure S19. Forest plots of ORs for the association of ADHD in pediatric patients with AD.

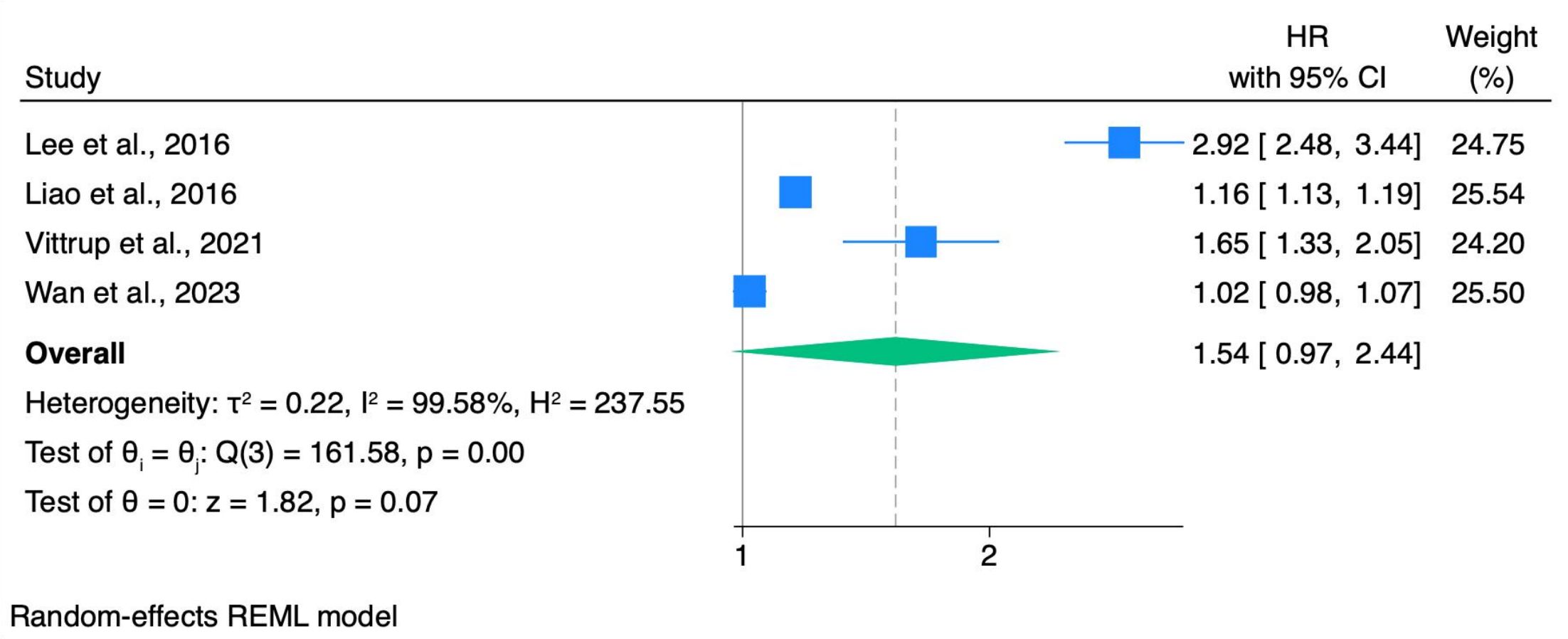

Figure S20. Forest plots of HRs for the association of ADHD in pediatric patients with AD.

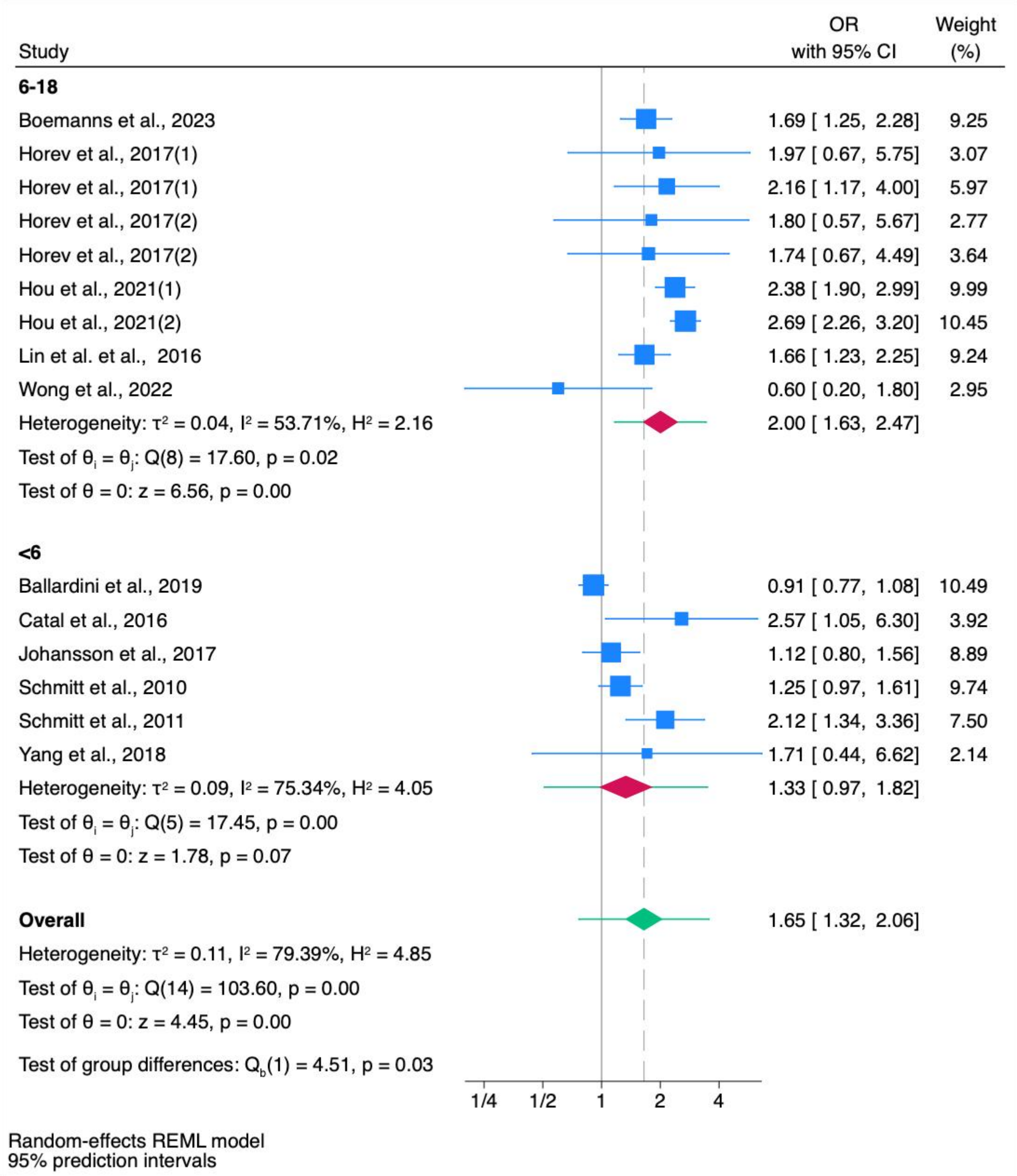

Figure S21. Exploratory subgroup analysis of ADHD association among pediatric patients with AD by age.

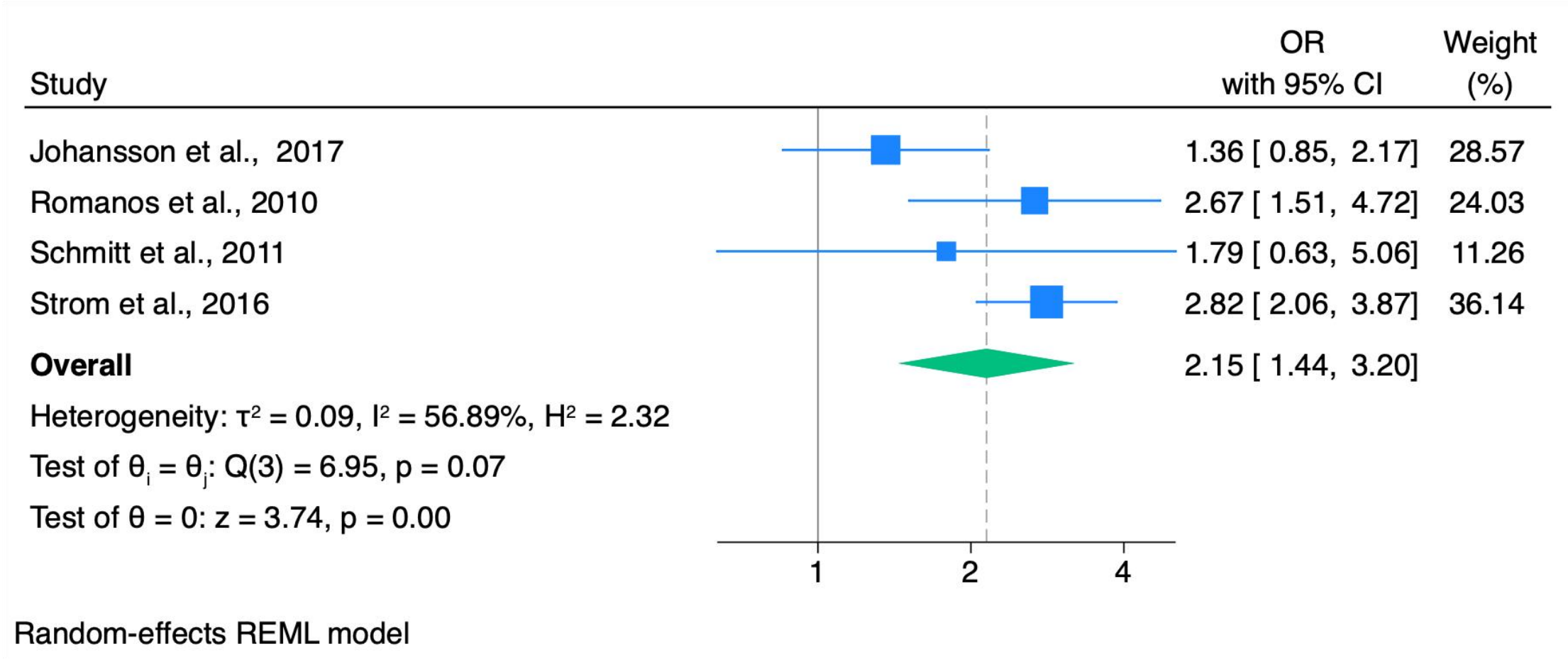

Figure S22. Exploratory pooled analysis of having sleep disturbance in relation to ADHD among pediatric patients with AD.

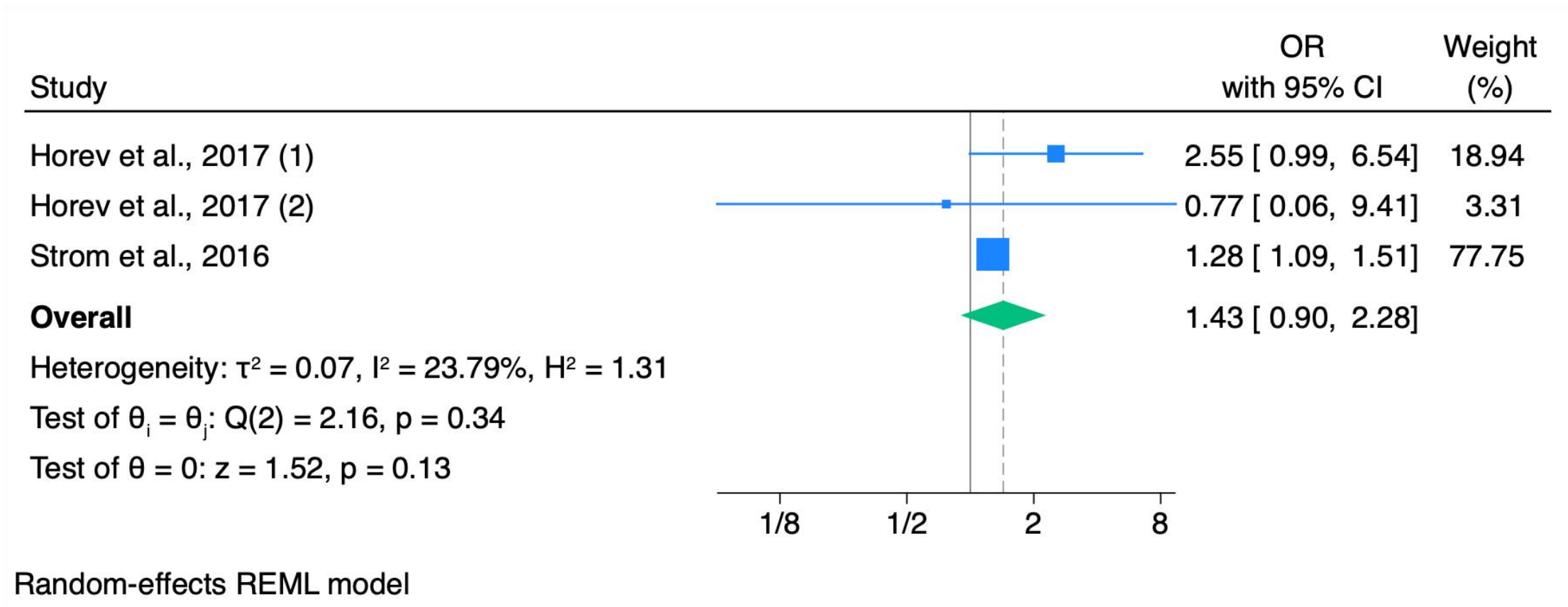

Figure S23. Exploratory pooled analysis of having obesity in relation to ADHD among pediatric patients with AD.

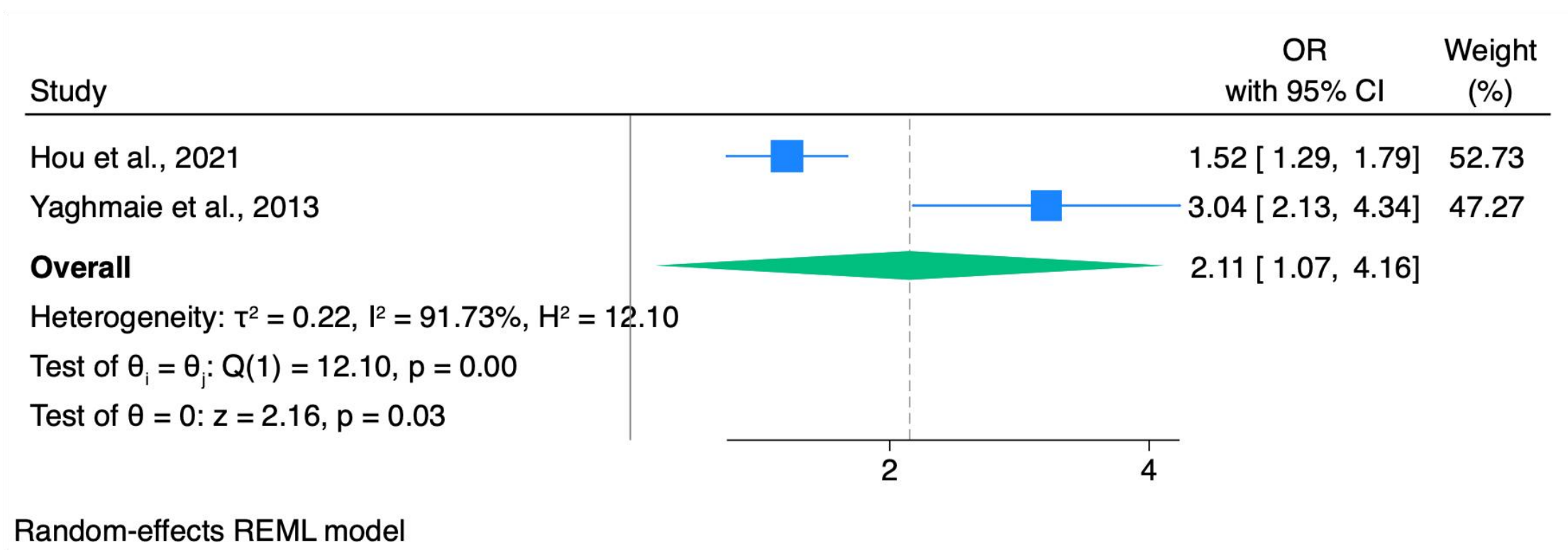

Figure S24. Forest plots of ORs for the association of ASD in pediatric patients with AD.

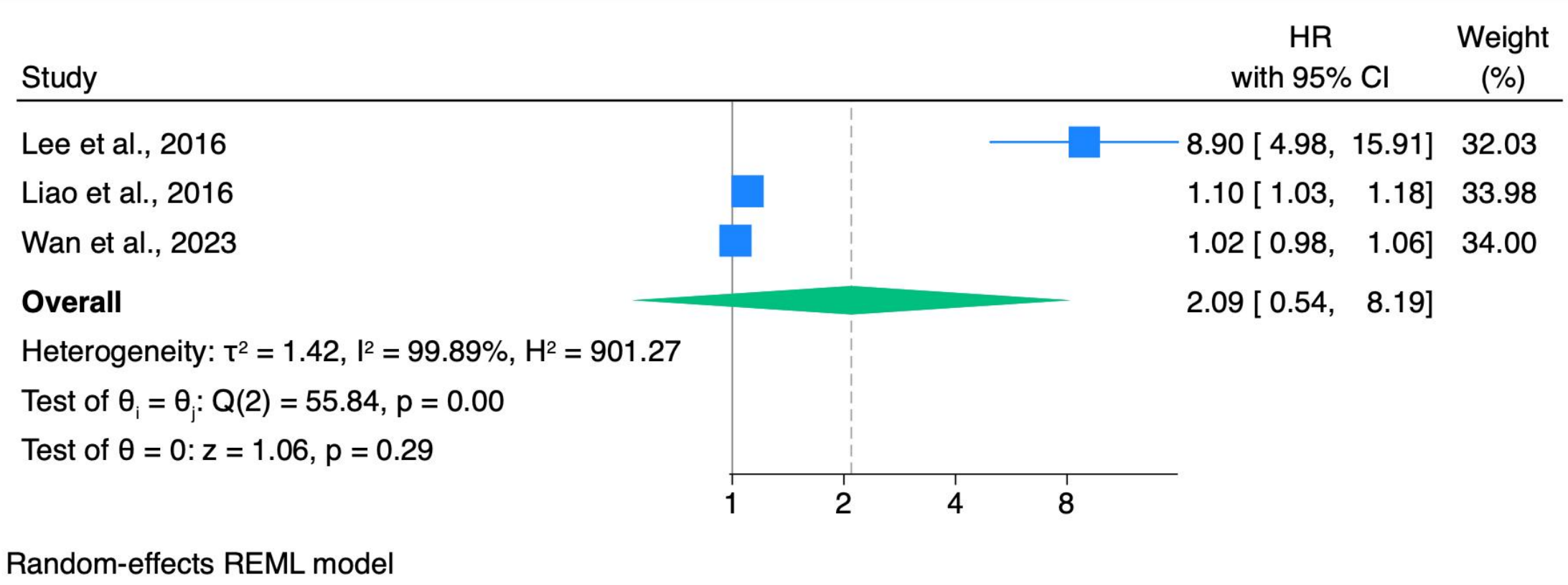

Figure S25. Forest plots of HRs for the association of ASD in pediatric patients with AD.

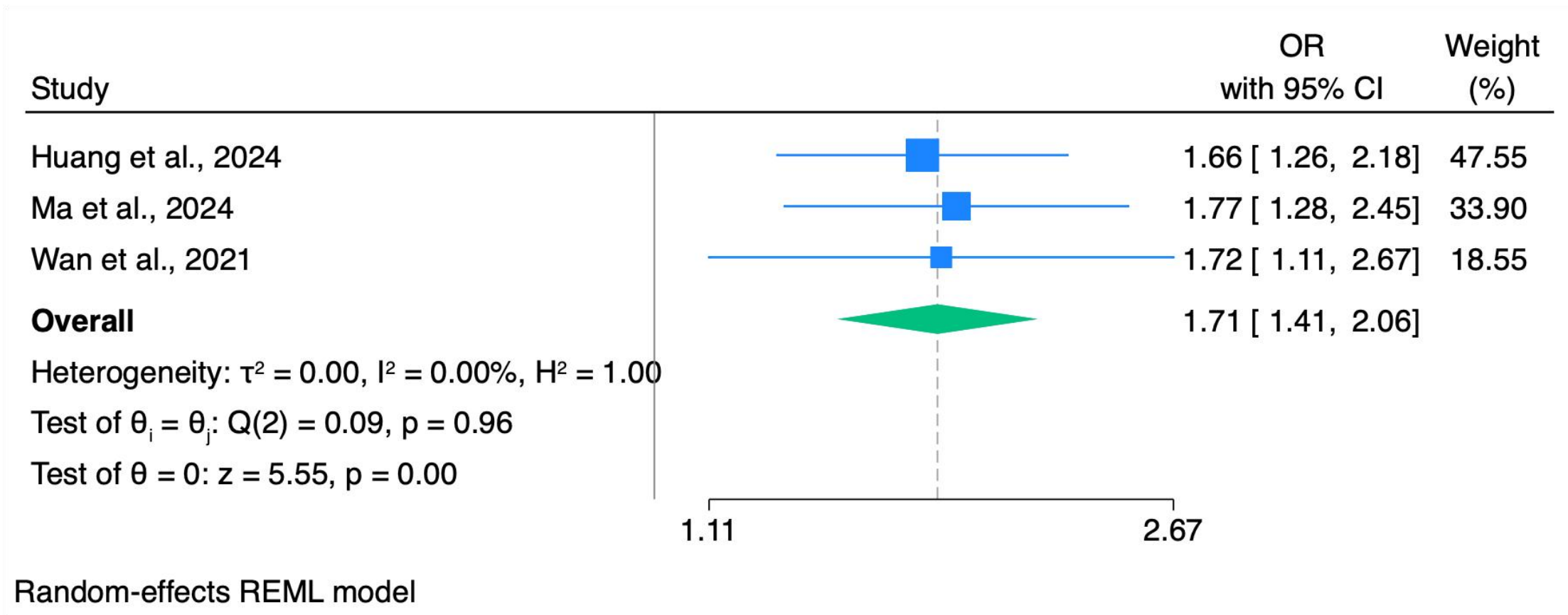

Figure S26. Forest plots for the association of learning disability in pediatric patients with AD.

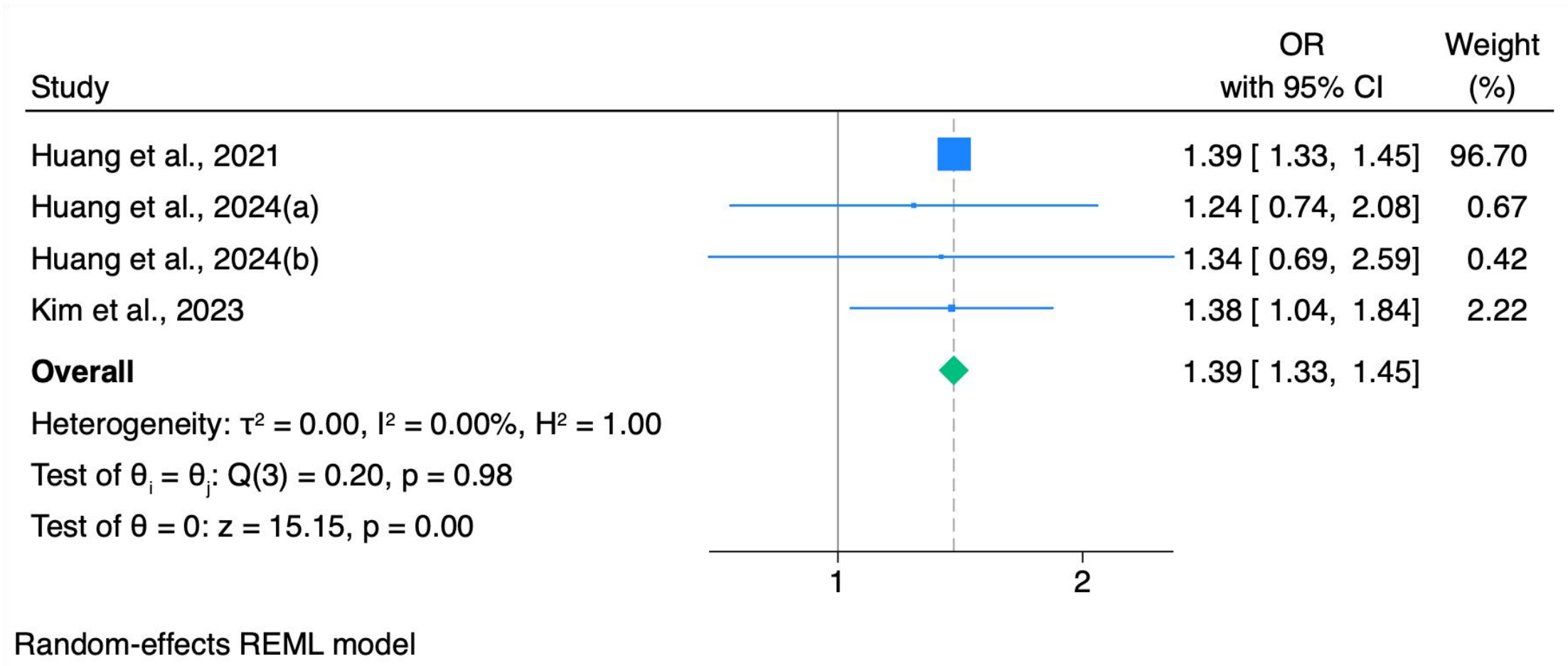

Figure S27. Forest plots for the association of intellectual disability in pediatric patients with AD.

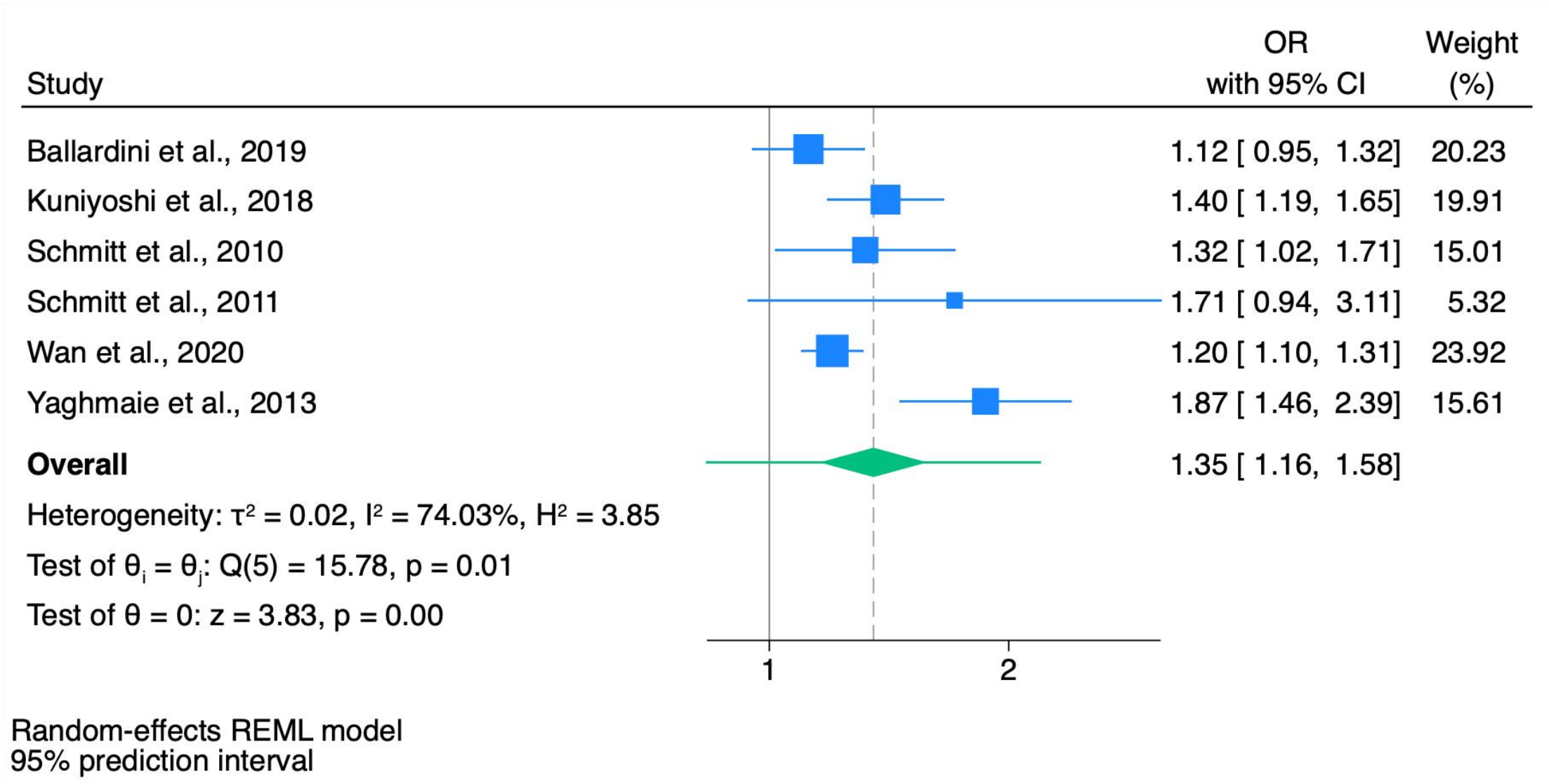

Figure S28. Forest plots for the association of conduct disorder in pediatric patients with AD.

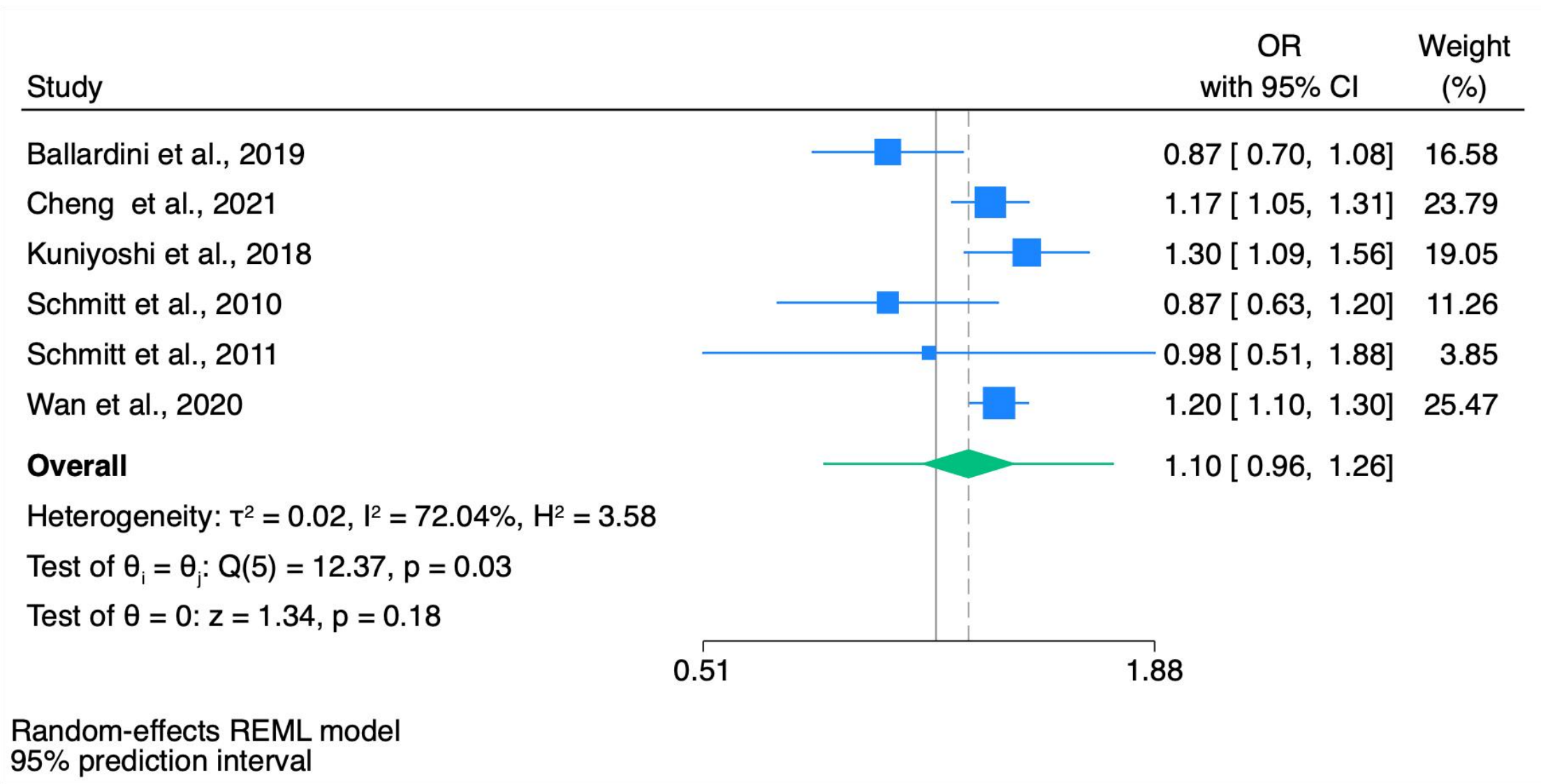

Figure S29. Forest plots for the association of peer problems in pediatric patients with AD.

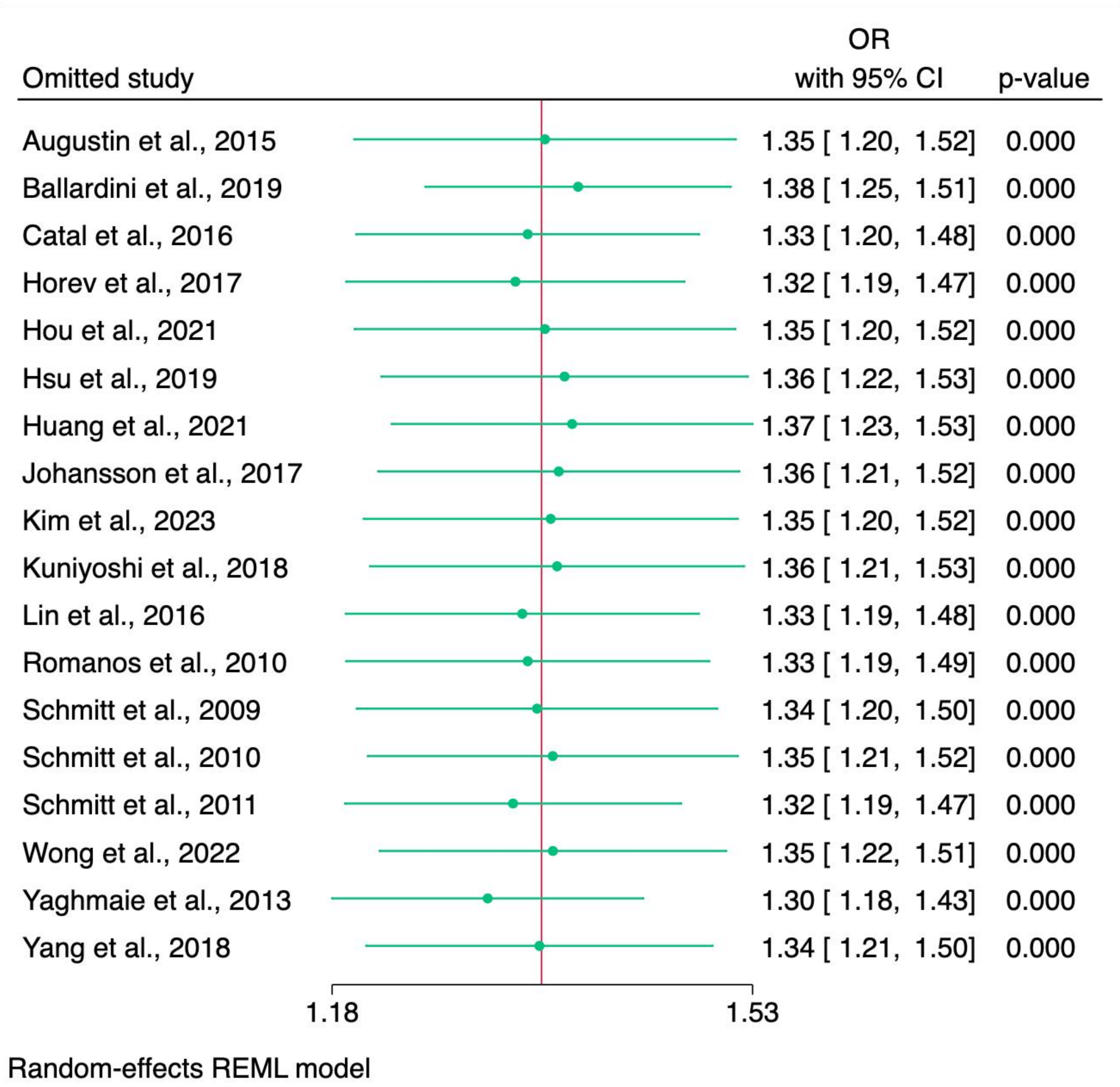

Figure S30. Leave-one-out sensitivity analysis of ORs for the association of ADHD in pediatric patients with AD.

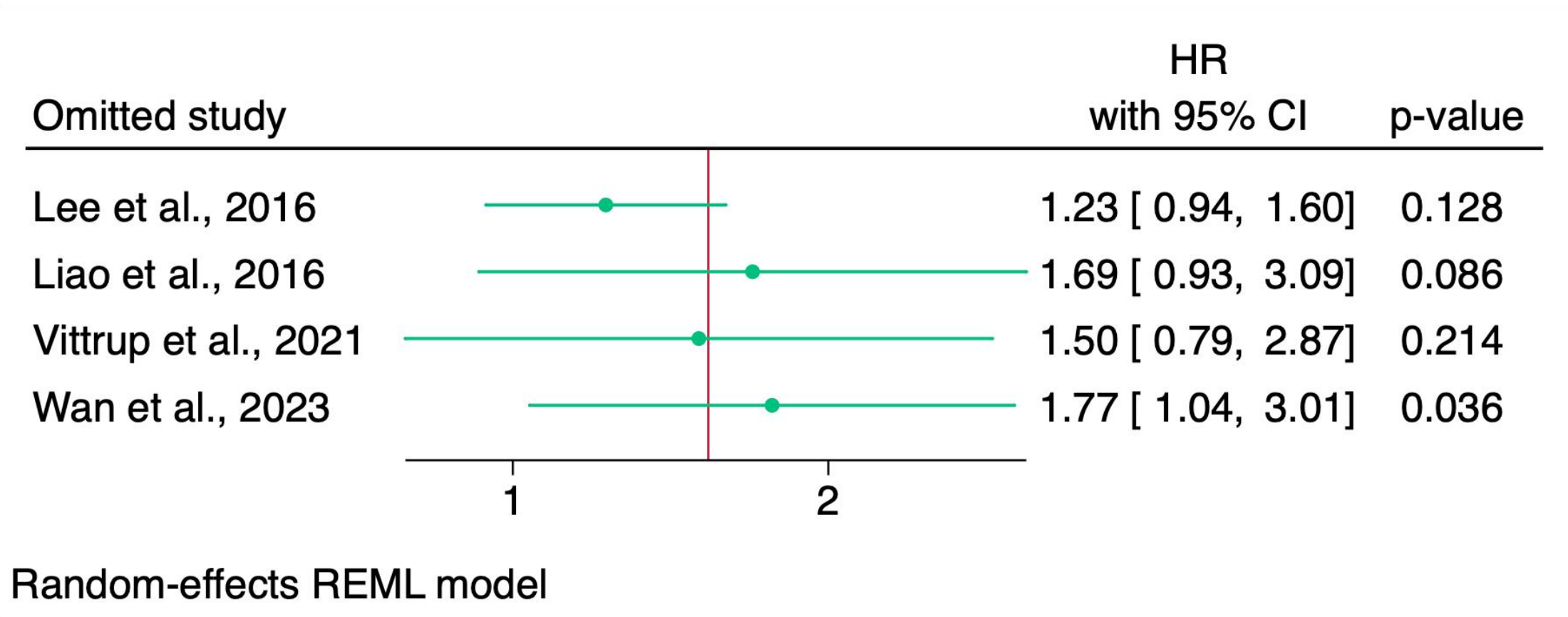

Figure S31. Leave-one-out sensitivity analysis of HRs for the association of ADHD in pediatric patients with AD.

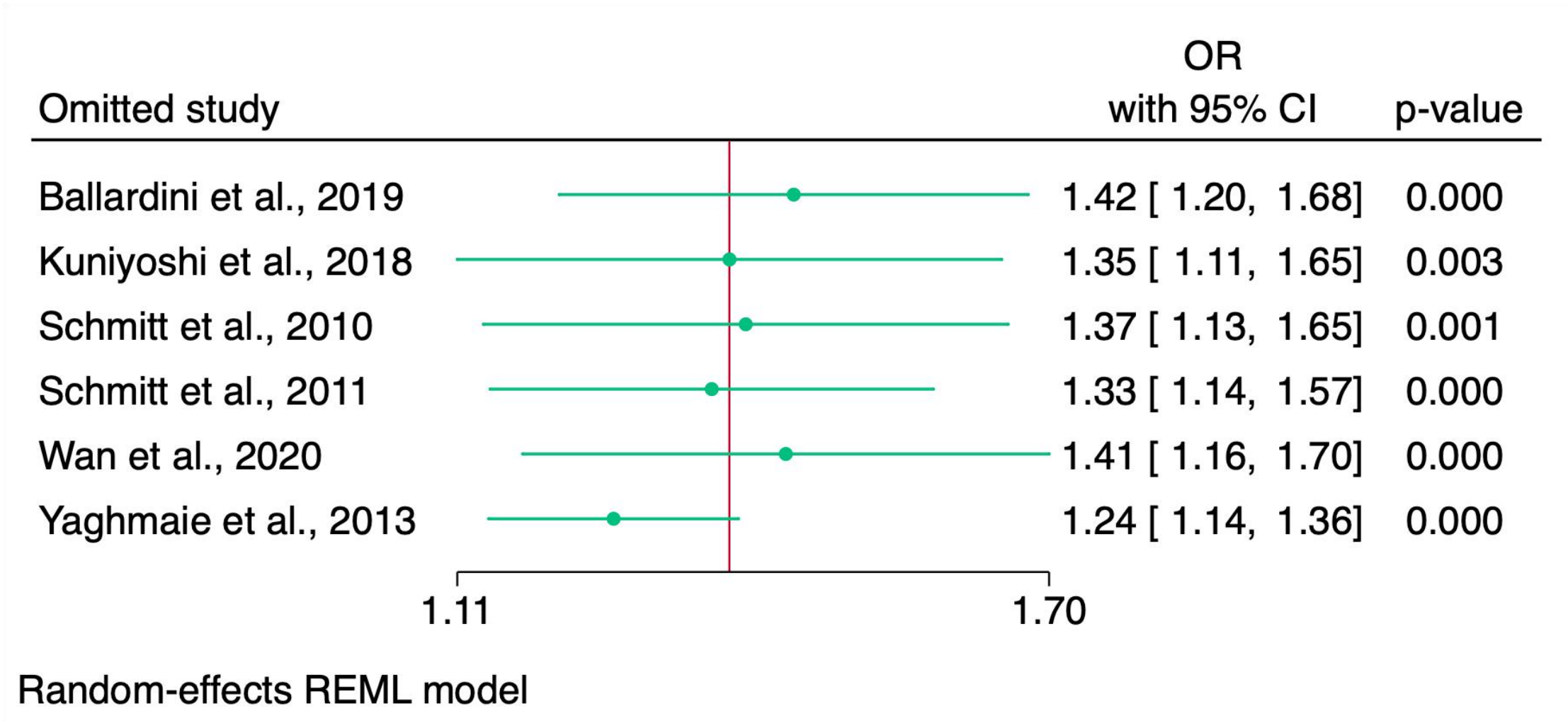

Figure S32. Leave-one-out sensitivity analysis for the association of conduct disorder in pediatric patients with AD.

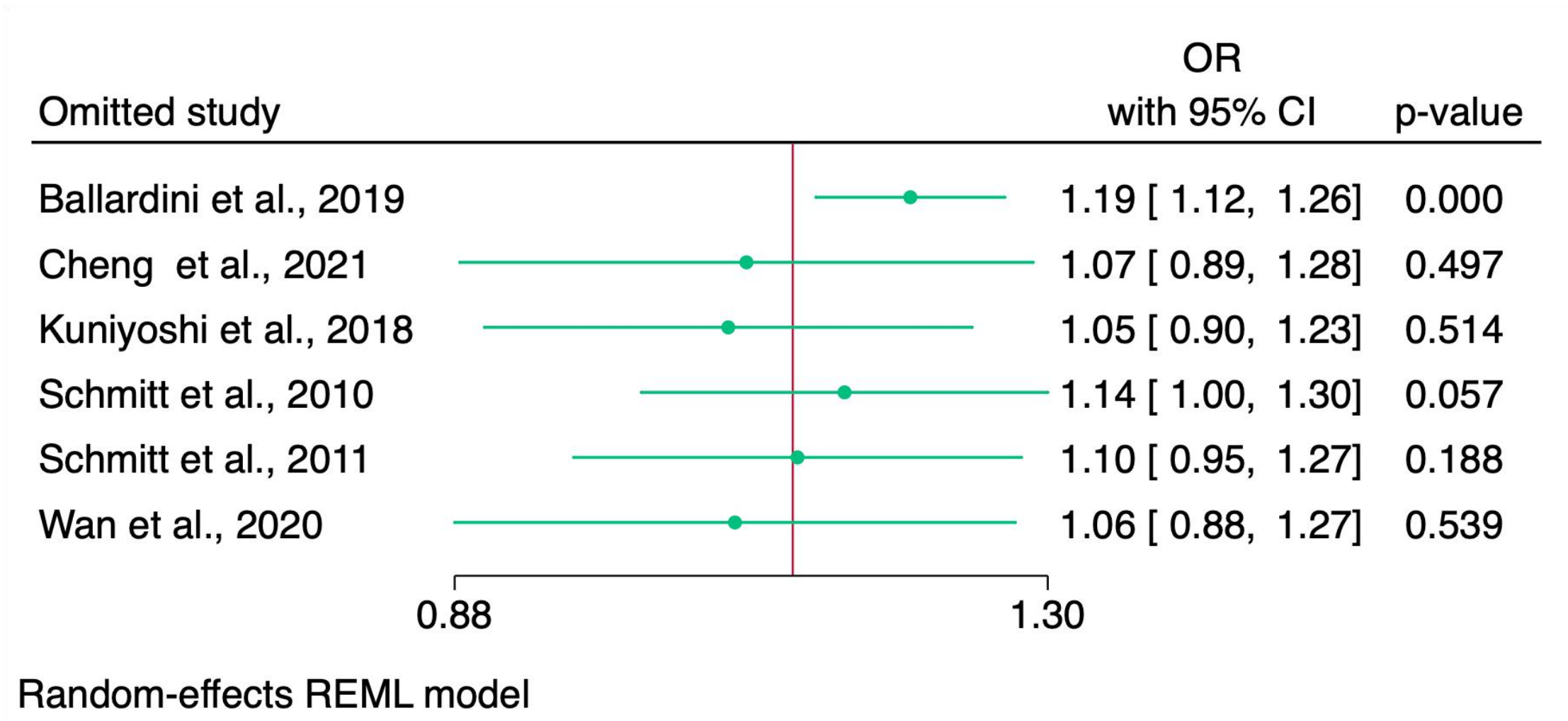

Figure S33. Leave-one-out sensitivity analysis for the association of peer problems in pediatric patients with AD.

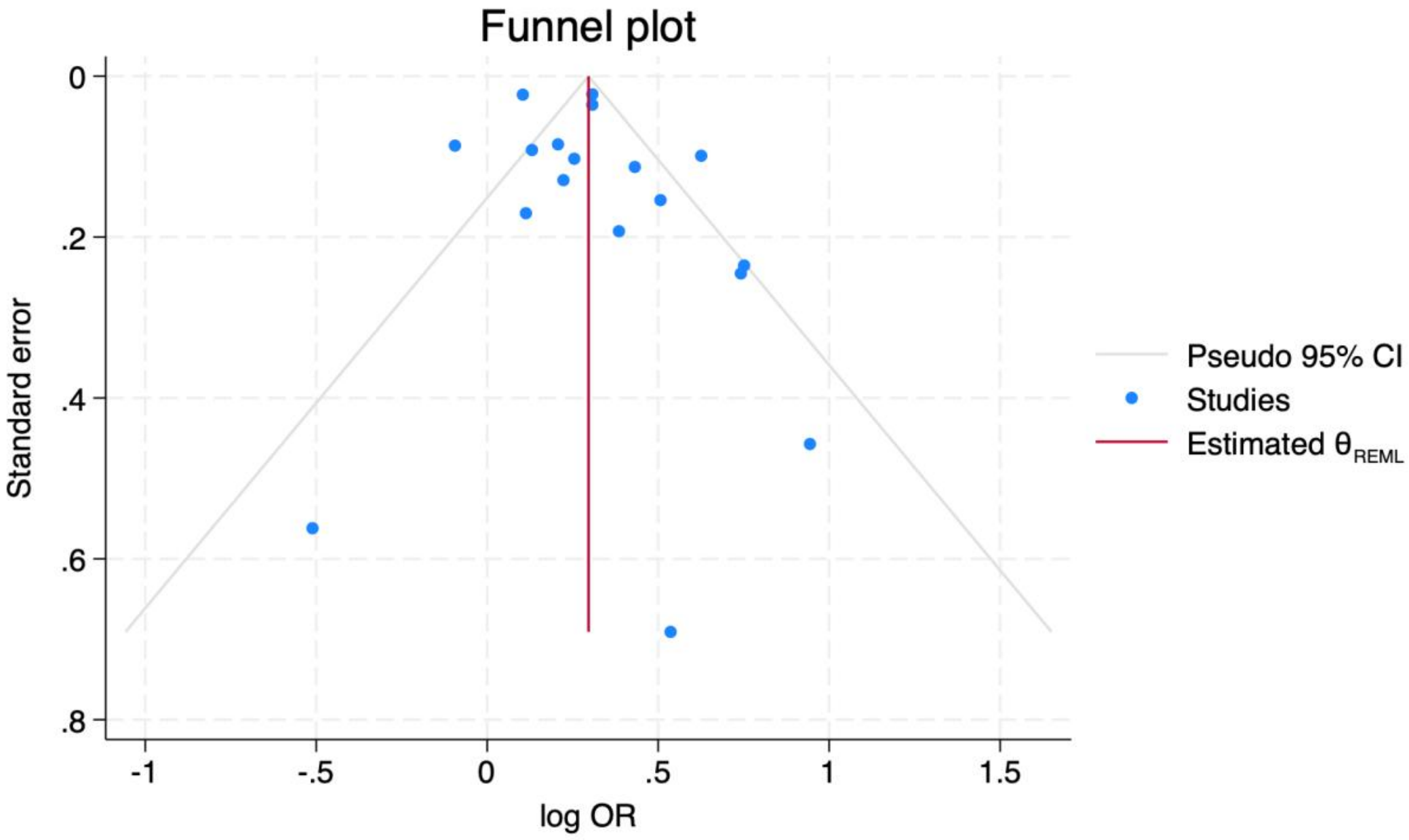

Figure S34. Funnel plot of ORs for the association of ADHD in pediatric patients with AD.
